# The Functional Availability of Arterial Kv7 Channels Is Suppressed Considerably by Large-Conductance Calcium-Activated Potassium Channels in 2- to 3-Month Old but Not in 10- to 15-Day Old Rats

**DOI:** 10.3389/fphys.2020.597395

**Published:** 2020-12-15

**Authors:** Dongyu Ma, Dina Gaynullina, Nadine Schmidt, Mitko Mladenov, Rudolf Schubert

**Affiliations:** ^1^European Center for Angioscience (ECAS), Research Division Cardiovascular Physiology, Medical Faculty Mannheim, Heidelberg University, Mannheim, Germany; ^2^Department of Cardiology, The First Affiliated Hospital of Anhui Medical University, Anhui, China; ^3^Faculty of Biology, M.V. Lomonosov Moscow State University, Moscow, Russia; ^4^Department of Fundamental and Applied Physiology, Russian National Research Medical University, Moscow, Russia; ^5^Institute of Biology, Faculty of Natural Sciences and Mathematics, Ss Cyril and Methodius University, Skopje, Macedonia; ^6^Department of Physiology, Institute of Theoretical Medicine, Medical Faculty, University of Augsburg, Augsburg, Germany

**Keywords:** arteries, ion channels, BK channel, Kv7 channel, ontogenesis, vascular smooth muscle

## Abstract

**Background:**

Voltage-gated potassium (Kv) channels, especially Kv7 channels, are major potassium channels identified in vascular smooth muscle cells with a great, albeit differential functional impact in various vessels. Vascular smooth muscle Kv7 channels always coexist with other K channels, in particular with BK channels. BK channels differ in the extent to which they influence vascular contractility. Whether this difference also causes the variability in the functional impact of Kv7 channels is unknown. Therefore, this study addressed the hypothesis that the functional impact of Kv7 channels depends on BK channels.

**Experimental Approach:**

Experiments were performed on young and adult rat *gracilis* and *saphenous* arteries using real-time PCR as well as pressure and wire myography.

**Key Results:**

Several subfamily members of Kv7 (KCNQ) and BK channels were expressed in saphenous and gracilis arteries: the highest expression was observed for BKα, BKβ1 and KCNQ4. Arterial contractility was assessed with methoxamine-induced contractions and pressure-induced myogenic responses. In vessels of adult rats, inhibition of Kv7 channels or BK channels by XE991 or IBTX, respectively enhanced arterial contractility to a similar degree, whereas activation of Kv7 channels or BK channels by retigabine or NS19504, respectively reduced arterial contractility to a similar degree. Further, IBTX increased both the contractile effect of XE991 and the anticontractile effect of retigabine, whereas NS19504 reduced the effect of retigabine and impaired the effect of XE991. In vessels of young rats, inhibition of Kv7 channels by XE991 enhanced arterial contractility much stronger than inhibition of BK channels by IBTX, whereas activation of Kv7 by retigabine reduced arterial contractility to a greater extent than activation of BK channels by NS19504. Further, IBTX increased the anticontractile effect of retigabine but not the contractile effect of XE991, whereas NS19504 reduced the effect of retigabine and impaired the effect of XE991.

**Conclusion:**

Kv7 and BK channels are expressed in young and adult rat arteries and function as negative feedback modulators in the regulation of contractility of these arteries. Importantly, BK channels govern the extent of functional impact of Kv7 channels. This effect depends on the relationship between the functional activities of BK and Kv7 channels.

## Introduction

In vascular smooth muscle cells (VSMCs), potassium channels evoke membrane hyperpolarization, reduce the entry of extracellular Ca^2+^ through voltage-dependent Ca^2+^ channels (VDCCs) and hence decrease their contractility (Nelson and Quayle, 1995; Coetzee et al., 1999; Jackson, 2000; Thorneloe and Nelson, 2005; Tykocki et al., 2017). VSMC contractility plays an essential role in the determination of vascular tone. The latter contributes considerably to the setting of blood pressure and the regulation of blood flow distribution to different organs and tissues.

Kv7 channels (Kv7.1–7.5), a subfamily of Kv channels (Kv1–12) (Wulff et al., 2009) are encoded by KCNQ (KCNQ1–5) genes. These channels play an important role in vasodilation and contribute to the negative feedback control of vasoconstriction. This role of Kv7 channels is based on their regulation by a number of factors including the membrane potential and signaling pathways involving PKC, PKA, PKG as well as G-protein βγ subunits (Mackie et al., 2008; Chadha et al., 2012; Stott et al., 2015a,b).

Another factor with potential regulatory impact on Kv7 channels are other K channels expressed in VSMCs (for an overview of VSMC K channels see Tykocki et al., 2017). Interestingly, a recent publication suggested a largely unexplored explanation for this phenomenon (Coleman et al., 2017). When VSMC membrane potential is close to the potassium equilibrium potential, driving force for potassium ions is small. Due to the small driving force, blockade of a particular potassium channel under these conditions will result in only a small change or in no change in membrane potential and vessel tone, hence a small functional role of this channel. A membrane potential close to the potassium equilibrium potential may be produced by another potassium channel underlying a dominant potassium conductance. Blockade of the latter potassium conductance will move the membrane potential away from the potassium equilibrium potential. This will result in a larger driving force for potassium and a larger functional role of the non-blocked K channels. Thus, the functional impact of a particular potassium channel will depend on the relative activities of the other potassium channels expressed, i.e., potassium channels will interact functionally.

Of note, a recent study showed an increased functional role of Kv7 channels in rat pulmonary arteries after inhibition of Kv1 and TASK-1 channels suggesting a functional interaction of Kv7 and Kv1/TASK-1 channels (Mondéjar-Parreño et al., 2020). A detailed investigation of this interaction was not the focus of the latter study.

BK channel expression has been shown in smooth muscle cells of all systemic arteries. These channels are involved in the negative feedback regulation of myogenic tone, they contribute to vasoconstriction and mediate vasodilation in almost all vessels studied (Nelson and Quayle, 1995; Jackson and Blair, 1998; Tykocki et al., 2017). Thus, BK channels seem to be a good candidate for the above mentioned dominant potassium conductance able to affect the functional role of other K channels including Kv7 channels. Indeed, BK channels have been shown to be the dominant negative feedback regulator of vasocontraction in adult rat saphenous arteries, whereas K_ATP_-, Kv1-, Kv2- and TASK-1 channels, each one tested for itself, are not involved in this response and Kir2- and Kv7-channels play only a small role (Shvetsova et al., 2019, 2020). However, none of the latter studies explores the interaction of K channels. Thus, the hypothesis was tested that the functional impact of Kv7 channels depends on BK channels. This interaction was studied initially for agonist-induced vasocontraction in rat saphenous arteries, where the role of different VSMC K channels has been described recently in detail (Shvetsova et al., 2019, 2020) and extended to the gracilis artery myogenic response, another important contractile stimulus. The gracilis artery was used because it possesses prominent myogenic reactivity (see data of the present manuscript), in contrast to the larger saphenous artery and belongs to the same vascular region as the saphenous artery.

## Materials and Methods

### Animals

The investigation conforms with the US Guide for the Care and Use of Laboratory Animals (Eighth edition, National Academy of Sciences, 2011) and approval for the use of laboratory animals in this study was granted by a governmental committee on animal welfare (I-17/17). Adult, 2- to 3-month old, and young, 10- to 15-days old, male Wistar rats (Janvier, France) were used. Food and water *ad libitum* was provided for the animals which were housed in a room with a controlled temperature and a 12-h light-dark cycle.

### Vessel Preparation

Rats were sacrificed under CO_2_ narcosis by decapitation. The lower extremity (limb) was quickly removed. It was placed in an ice-cold physiological saline solution composed of (in mM): 145 NaCl, 4.5 KCl, 1.2 NaH_2_PO_4_, 0.1 CaCl_2_, 1.0 MgSO_4_, 0.025 EDTA, 5 HEPES (pH 7.4). The gracilis artery and the saphenous artery were isolated by removing all surrounding skeletal muscle and connective tissue. Further experiments were performed on small rings 2 mm in length.

### Isobaric Mounting of Gracilis Arteries

The gracilis artery was mounted on two glass pipettes which were fixed in the experimental chamber of a pressure myograph (DMT 201CM, Danish Myotechnology, Denmark). This chamber contained experimental solution (physiological saline solution, PSS) consisting of (in mM) 146 NaCl, 4.5 KCl, 1.2 NaH_2_PO_4_, 1.0 MgSO_4_, 1.6 CaCl_2_, 0.025 EDTA, 5.5 glucose and 5 HEPES at pH 7.4. The microscope image of the vessel was viewed with a CCD camera and digitized by a frame-grabber card (Hasotec, Germany). Diameter changes were measured continuously at a sampling rate of 0.5 Hz with use of a custom-made program (Fischer et al., 1996). Of note, the internal diameter is the most appropriate parameter to characterize the functional state of the vessels. However, when we tested the combined effect of IBTX and XE991 a strong constriction occurred. Under these conditions the connective tissue remaining even after careful dissection (more rigorous removal of the connective tissue led to a loss of myogenic reactivity) masked the inner diameter; in fact the inner diameter could not be determined any more. Thus, the external diameter had to be used to assess myogenic reactivity. Vessels were kept at a pressure of 80 mmHg without any luminal flow at a temperature of 37°C. Leaking vessels were discarded at any stage of the experiment to ensure complete non-flow conditions. After development of a spontaneous myogenic tone vessel viability was tested by application of 10^–5^ M methoxamine to test smooth muscle cell function and 10^–6^ M acetylcholine to test endothelial cell function. All vessels were exposed to calcium-free solution to determine the fully relaxed diameter at 80 mmHg at the end of the experiment. The fully relaxed diameter of the vessels in this study was in the range from 210 to 235 μm. All diameter values were normalized to the diameter of the same fully relaxed vessel at 80 mmHg in calcium-free solution. Normalization was done in order to eliminate variability due to differences in the size of different vessels.

### Isometric Mounting of Saphenous Arteries

The isolated saphenous artery was mounted in a wire myograph (model 620M, Danish Myotechnology, Denmark) on two 40 μm wires. Isometric tension was recorded where data acquisition and analysis was performed using Labchart (ADInstruments, United States). The vessels were stretched to their optimal lumen diameter (90% of the diameter they would have at a transmural pressure of 100 mmHg (Mulvany and Halpern, 1977). They were placed in PSS consisting of (in mM) 120 NaCl, 4.5 KCl, 1.2 NaH_2_PO_4_, 1.0 MgSO_4_, 1.6 CaCl_2_, 0.025 EDTA, 5.5 glucose, 26 NaHCO_3_, and 5 HEPES at pH 7.4 oxygenated with carbogen (95% O_2_ and 5% CO_2_) at 37°C. Viability of the vessels was tested by application of 10^–5^ M methoxamine to test smooth muscle cell function and of 10^–5^ M acetylcholine after preconstriction with 10^–7^ M methoxamine to test endothelial cell function. Vessel tension was normalized to the tension developed in response to 10^–5^ M methoxamine applied directly after the viability test. This was done in order to eliminate variability due to differences in the contractility of different vessels. Special care was taken to carefully match vessel tension before the intervention to be able to compare vessel responses to different interventions. Thus, when 4 groups of vessels with different interventions (e.g., application of IBTX, NS19504, IBTX + NS19504, control = application of vehicle) were compared, it was ensured that the averaged methoxamine-induced concentration-response relationships (or the pressure-diameter relationships in isobaric preparations) of these 4 vessel groups obtained directly after the 10^–5^ M methoxamine-test and before the addition of the above mentioned substances did not differ (see for example Schmid et al., 2018, Supplementary Figure 1A).

### Functional Removal of the Endothelium

The endothelium was removed from all vessels studied in pressure or wire myographs. In isobaric experiments this was done by passing an air bubble through the lumen of the vessel. In isometric experiments the endothelium was disrupted mechanically using a rat whisker. Functional removal of the endothelium was considered successful when acetylcholine-induced vasodilation was absent during the viability test.

### Real-Time PCR

Vessels (saphenous and gracilis arteries) were isolated in two groups, with endothelium(E+) and without endothelium(E−) as described above. They were cut into small pieces and homogenized for 3 min at 30 Hz in the TissueLyser II (Qiagen). Total RNA was isolated using the “RNeasy Mini-Kit” (Qiagen) according to the manufacturer instructions. Optional On-Column DNase Digestion using the RNase-Free DNase Set (Qiagen) was performed as described in the manufacturer instructions. In the final step, RNA was collected from the affinity column using 30 μl RNase-free H2O, which was passed twice over the column. RNA concentration was determined on the Tecan infinite 200 PRO.

Reverse transcription to cDNA was performed by Mastercyler (Gradient 5333, Eppendorf) using 5 × Reaction Buffer (Thermo scientific), dNTP Mix (10 nM each, Thermo scientific), RevertAid H Minus ReverseTranscriptase (Thermo scientific), and Random Primers (Invitrogen) according to the manufacturer’s standard protocol. All samples were diluted to a starting concentration of 5 ng RNA per μL of reaction.

Samples were quantified with real-time PCR using SensiFAST^TM^ SYBR No-ROX Kit, 2× (Bioline, Cat# BIO-98020) on Light Cycler 480 (Roche) according to the manufacturer’s standard protocol. Primers were purchased or self-designed and ordered from Eurofins. Amplicon context sequence and amplicon length can be found on the Bio-Rad Homepage^1^ in accordance with the Guidelines for Minimum Information for Publication of Quantitative Digital PCR Experiments (MIQE) guidelines (Huggett et al., 2013). The following genes (with their Sequence) have been tested: Kcnq1 (ffw: GGCTCTGGGTTTGCACTG, rev: CATAGCA CCTCCATGCAGTC), Kcnq2 (ffw: ACACAGACTCAGACCTC TGCAC, rev: AGCCCAACCCAGAATCACTTCC), Kcnq3 (ffw: GCTAGGGACCGGAGCCGACA, rev: CCCCTCGGTCTCTCC AGGGC), Kcnq4 (ffw: CCCCGCTGCTCTACTGAG, rev: ATG ACATCATCCACCGTGAG), Kcnq5 (ffw: GATGCCAGTGTGA CGTGTCCGTGG, rev: CCTTTCCGAGGACCTGCTGGTAG), rBKalpha (ffw: AAACAAGTAATTCCATCAAGCTGGTG, rev: CGTAAGTGCCTGGTTGTTTTGG), rBKbeta1 (ffw: ACCAAT CTCTTCTGCACAGCAGC, rev: AGAGCTGTGACTGGCAGT TCCTT), eNOS (ffw: GGATTCTGGCAAGACCGATTAC, rev: GGTGAGGACTTGTCCAAACACT), Hmbs (ffw: GCGGAAG AAAACGGCTCAATG, rev: AGCATCGCTACCACAGTGTC), Gapdh (ffw: CACCAGCATCACCCCATTT; rev: CCATCAAGG ACCCCTTCATT). Hmbs was used as reference gene for the expression analysis of endothelium-denuded and endothelium-intact vessels; Gapdh was used as reference gene for the expression analysis of vessels from young and adult rats. For each reaction of 20 μL, a volume of 2 μL cDNA was used (as an equivalent of 10 ng starting RNA). At the end of reaction, melting curves were checked by use of the Light Cycler 480 software to ensure the specificity of qPCR products. The LinRegPCR program (Academic Medical Center, Netherlands) was used to analyze the real-time PCR data. mRNA expression levels were calculated as E^–Ct^, where E is the primer efficiency and Ct is the cycle number on which product fluorescence rose above the threshold level. Primer efficiency was determined for every primer pair using LinReg. These expression values were related to the expression values of the housekeeping gene detected in the same sample. Expression data from vessels from young and adult rats were normalized to the mean of the expression of vessels from adult rats.

### Selection Criteria for BK and Kv7 Channel Blocker and Opener Concentrations

Iberiotoxin (IBTX) has widely been employed as a BK channel inhibitor starting from 1990 (Galvez et al., 1990). It has been used since then in an uncountable number of reports and proven to be a specific BK channel blocker. IBTX was reported to have an IC_50_ of 1.7 nM (Tykocki et al., 2017). Since a complete block of BK channels was desired, 100 nM IBTX was employed.

NS19504 is a relatively new opener of BK channels with an EC_50_ of 11 μM for bladder smooth muscle BK channels (determined with a Tl^+^ assay under conditions differing quite a lot compared to the conditions employed in experiments on intact organs) and 640 nM for the inhibition of bladder spontaneous contractile activity (Nausch et al., 2014). Of note, the inhibition of spontaneous bladder activity by NS19504 was not blocked completely by IBTX pointing to a non-specific effect of this new compound at higher concentrations. Data on the effect of NS19504 on vascular smooth muscle BK channels are not available. Therefore, we studied the effect of NS19504 on rat arteries in detail (see “Results” section). NS19504 at 6^∗^10^–6^ M was selected as the most appropriate concentration, i.e., the concentration that produces the largest but still selective effect on vascular contractility.

**FIGURE 1:**
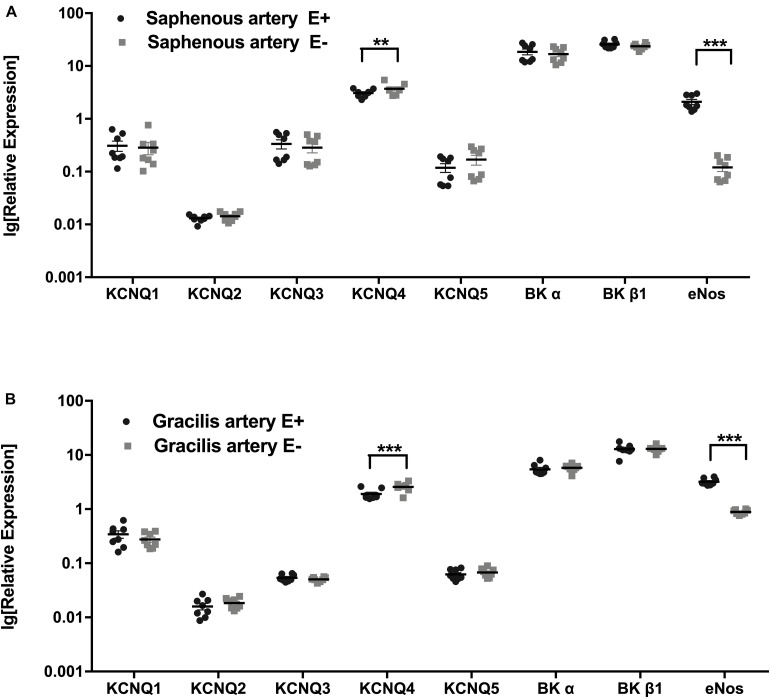
Expression of KCNQ and BK channel genes. **(A)** Relative expression profile (related to Hmbs) of KCNQ and BK channel genes in Saphenous artery with endothelium (E+) and without endothelium (E–). **(B)** Relative expression profile of KCNQ and BK channel genes in gracilis artery with endothelium (E+) and without endothelium (E–). *n* = 8; **p < 0.01; ***p < 0.001.

XE991 is a well-known blocker of Kv7 channels with an IC_50_ of 5.5 μM determined for Kv7.4 channels expressed in HEK cells (Søgaard et al., 2001; Gollasch et al., 2018). Kv7.4 channels are the most prominent isoform expressed in the vessels studied in the present investigation (see Figure 1 of the present manuscript for saphenous and gracilis arteries and Figure 3 in Zavaritskaya et al., 2013 for gracilis arteries). Taking into account the considerable differences in experimental conditions between patch-clamp studies on channels or currents and myography experiments on intact vessels, this IC_50_ value was used as a first orientation. Additionally, recent data were considered where we found that in an intact vessel preparation the effect of a Kv7 channel opener was inhibited by 3^∗^10^–6^ M XE991 and 10^–5^M XE991 to the same degree (Zavaritskaya et al., 2020). In addition, unpublished data on gracilis arteries showed that the effect of 3^∗^10^–5^ M XE991 on spontaneous myogenic tone was smaller compared to 3^∗^10^–6^ M XE991 and 10^–5^M XE991 pointing to an unspecific effect at this high concentration. Thus, in order to ensure the largest but still selective inhibitory effect of XE991, 3^∗^10^–6^ M was employed.

Retigabine is a well described opener of Kv7 channels with EC_50_ values between 1 and 5 μM for expressed Kv7.4 channels (Gollasch et al., 2018). We used previous data from intact vessel preparations to select the most appropriate concentration of retigabine. Retigabine at concentrations > 3^∗^10^–5^ M was able to dilate KCl-preconstricted vessels (Zavaritskaya et al., 2013) suggesting an effect independent of K channels at this concentration. The present study shows that the effect of retigabine at 3^∗^10^–5^ M could be abolished by XE991. Therefore, this concentration of retigabine was selected.

### Drugs and Chemicals

Methoxamine, acetylcholine as well as the salts for the solutions were obtained from Sigma (Germany). Iberiotoxin was purchased from Alomone Labs (Israel). NS19504, Retigabine and XE991 were obtained from Tocris (United Kingdom).

### Data Analysis and Statistics

The anti-contractile or contractile effect of a certain substance was calculated as the area between the methoxamine-induced concentration-response relationships or the pressure-diameter relationships obtained in the absence and in the presence of this substance (see also Schmid et al., 2018).

All values are given as mean ± SEM; n is the number of animals tested. Statistical analysis was performed using GraphPadPrism 6.0 (GraphPad Software, Inc.) employing ANOVA or paired *t*-tests as appropriate and a value of *p* < 0.05 was considered statistically significant.

## Results

### Expression of Kv7 and BK Channel Genes in Arteries of Adult Rats

The expression profiles of the subunits of KCNQ and BK channel genes in saphenous arteries were similar to those in gracilis arteries: the highest expression was observed for BKα, BKβ1 and KCNQ4, followed by KCNQ1, KCNQ3 and KCNQ5 at considerably lower expression levels. The lowest expressed gene was KCNQ2 (Figures 1A,B).

Expression profiles were compared for vessels without (E−) and with (E+) endothelium. Successful removal of the endothelium was verified by a substantially lower expression of the endothelial marker eNOS in vessels without endothelium (Figures 1A,B). In both vessels, no differences in KCNQ and BK channel genes were detected between vessel without and with endothelium, except for KCNQ4, which was expressed at a slightly lower level in vessels with endothelium (Figures 1A,B).

### Functional Interaction of Kv7 and BK Channels in Arteries of Adult Rats

The functional interaction of Kv7 and BK channels was studied by addition of their respective blockers and activators. Specifically, XE991 was used as a selective blocker of Kv7 channels, retigabine as a selective activator of Kv7 channels; iberiotoxin (IBTX) was used as a selective blocker of BK channels, and NS19504 as a novel activator of BK channels (for more details on the selection criteria for the BK and Kv7 channel blocker and opener concentrations see methods section).

Kv7 and BK channel functional interaction was studied using two approaches addressing different mechanisms regulating vessel contractility. Firstly, isometric preparations of saphenous arteries, a larger artery showing stable vasoconstrictor-induced contractions without spontaneous basal tone, were employed. Contractility was tested over a wide range of vessel tension achieved by application of methoxamine at concentrations between 10^–8^ M and 10^–5^ or 10^–4^M. Secondly, isobaric preparations of gracilis arteries, a smaller, myogenically active artery, were used. Contractility was tested over a wide pressure range between 10 and 120 mmHg.

#### Effect of Retigabine and XE991 on Arterial Contraction

Retigabine at 3^∗^10^–5^M strongly attenuated methoxamine-induced contractions, i.e., demonstrated an anti-contractile effect, whereas for XE991 at 3^∗^10^–6^M an effect was not detected in this experiment (Supplementary Figures 1A,B). In the presence of retigabine, however, XE991 enhanced methoxamine-induced contractions compared to these contractions in the presence of retigabine alone, i.e., demonstrated a contractile effect (Supplementary Figure 1B). Of note, in the presence of XE991 retigabine was without effect on methoxamine-induced contractions compared to these contractions in the presence of XE991 alone (Supplementary Figure 1B). Thus, the anti-contractile effect of retigabine was abolished by XE991 (Supplementary Figure 1C). Such an effect of XE991 was also observed when contractility was assessed based on the myogenic response of gracilis arteries (Supplementary Figures 2A–C).

#### Effect of NS19504 and IBTX on Arterial Contraction

Because there is no agent commonly recognized and confirmed as a selective activator of vascular smooth muscle BK channels, NS19504 as a novel candidate was tested in more detail, i.e., at three concentrations (3^∗^10^–6^M, 6^∗^10^–6^M, 10^–5^M).

NS19504 at 3^∗^10^–6^M attenuated methoxamine-induced contractions, while IBTX at 10^–7^M enhanced it (Supplementary Figures 3A,B,E1). In the presence of NS19504 IBTX also enhanced methoxamine-induced contractions compared to these contractions in the presence of NS19504 alone (Supplementary Figure 3E1). Of note, in the presence of IBTX NS19504 was without effect on methoxamine-induced contractions compared to these contractions in the presence of IBTX alone (Supplementary Figure 3E1). Thus, the anti-contractile effect of 3^∗^10^–6^M NS19504 was abolished by IBTX (Supplementary Figure 3F1). Such an effect of IBTX was also observed when contractility was assessed based on the myogenic response of gracilis arteries (Supplementary Figures 4A–C).

NS19504 at 6^∗^10^–6^M attenuated methoxamine-induced contractions, while IBTX at 10^–7^M enhanced it (Supplementary Figures 3A,C,E2). In the presence of NS19504 IBTX also enhanced methoxamine-induced contractions compared to these contractions in the presence of NS19504 alone (Supplementary Figure 3E2). Of note, in the presence of IBTX NS19504 was without effect on methoxamine-induced contractions compared to these contractions in the presence of IBTX alone (Supplementary Figure 3E2). Thus, the anti-contractile effect of 6^∗^10^–6^M NS19504 was abolished by IBTX (Supplementary Figure 3F2).

NS19504 at 10^–5^M attenuated methoxamine-induced contractions, while IBTX at 10^–7^M enhanced it (Supplementary Figures 3A,D,E3). In the presence of NS19504 IBTX also enhanced methoxamine-induced contractions compared to these contractions in the presence of NS19504 alone (Supplementary Figure 3E3). In the presence of IBTX NS19504 attenuated methoxamine-induced contractions compared to these contractions in the presence of IBTX alone (Supplementary Figure 3E3). Thus, the anti-contractile effect of 10^–5^M NS19504 was reduced but not abolished by IBTX (Supplementary Figure 3F3).

#### The Functional Impact of Kv7 Channels Increases After Blocking BK Channels

**FIGURE 2:**
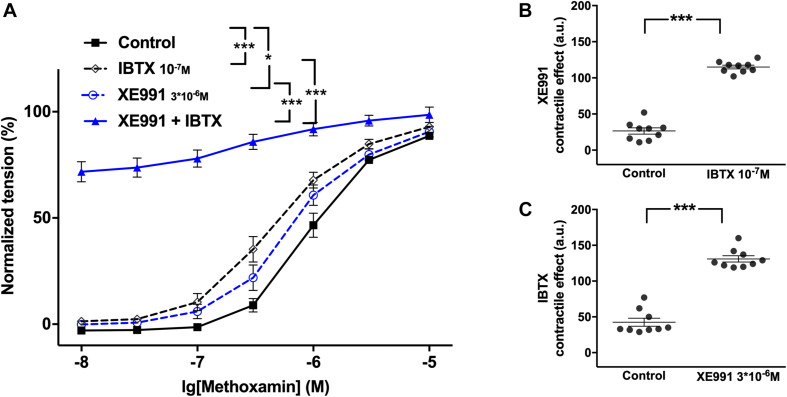
Effect of XE991 and IBTX on methoxamine-induced contractions of the saphenous artery. **(A)** Normalized tension of saphenous arteries with different methoxamine concentrations in the absence of potassium channel active agents (Control), in the presence of IBTX (IBTX 10^–7^M), in the presence of XE991 (XE991 3*10^–6^M) and in the combined presence of XE991 and IBTX (XE991 + IBTX). **(B)** XE991 contractile effect in the absence (Control) and presence of IBTX (IBTX 10^–7^M). **(C)** IBTX contractile effect in the absence (Control) and presence of XE991 (XE991 3*10^–6^M). *n* = 12; *p < 0.05, ***p < 0.001.

XE991 at 3^∗^10^–6^M as well as IBTX at 10^–7^M enhanced methoxamine-induced contractions (Figure 2A). In the presence of IBTX, XE991 also enhanced methoxamine-induced contractions compared to these contractions in the presence of IBTX alone (Figure 2A). Further, in the presence of XE991, IBTX enhanced methoxamine-induced contractions compared to these contractions in the presence of XE991 alone (Figure 2A). Thus, the contractile effect of XE991 was enhanced by IBTX (Figure 2B), and the contractile effect of IBTX was enhanced by XE991 (Figure 2C). Such effects of IBTX and XE991 were also observed when contractility was assessed based on the myogenic response of gracilis arteries (Supplementary Figures 5A–C).

**FIGURE 3:**
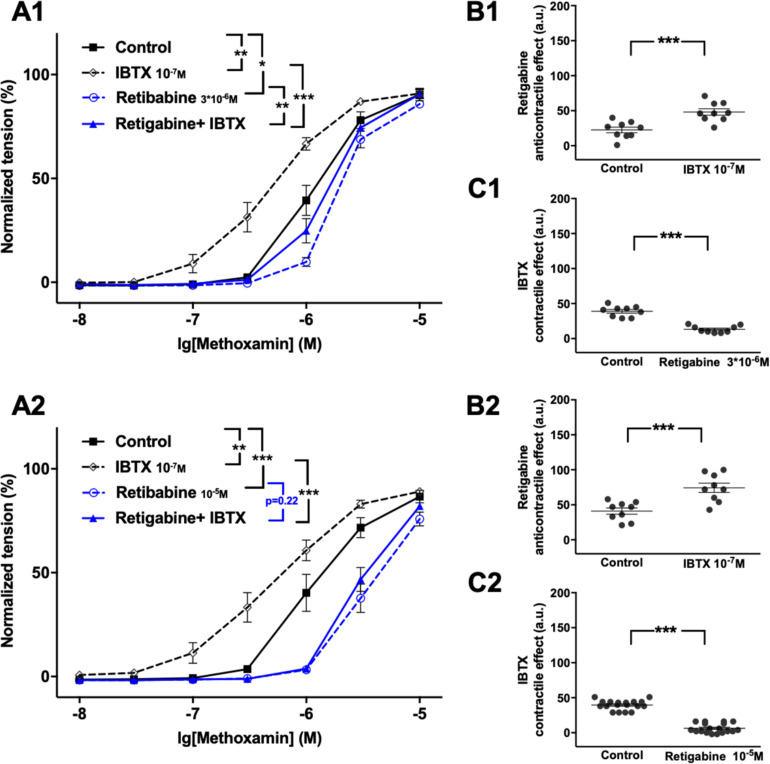
Effect of retigabine and IBTX on methoxamine-induced contractions of the saphenous artery. **(A1,A2)** Normalized tension of saphenous arteries with different methoxamine concentrations in the absence of potassium channel active agents (Control), in the presence of IBTX (IBTX 10^–7^M), in the presence of retigabine (**A1**: Retigabine 3*10^–6^M; **A2**: Retigabine 10^–5^M) and in the combined presence of retigabine and IBTX (Retigabine + IBTX). **(B1,B2)** Retigabine anti-contractile effect in the absence of (Control) and presence of IBTX (IBTX 10^–7^M). **(C1,C2)** IBTX contractile effect in the absence of (Control) and presence of retigabine (**C1**: Retigabine 3*10^–6^M, **C2**: Retigabine 10^–5^M). n1 = 9, n2 = 9; *p < 0.05, **p < 0.01; ***p < 0.001.

Further, retigabine at 3^∗^10^–6^M attenuated methoxamine-induced contractions, whereas IBTX at 10^–7^M enhanced methoxamine-induced contractions (Figure 3A1). In the presence of retigabine IBTX also enhanced methoxamine-induced contractions compared to these contractions in the presence of retigabine alone (Figure 3A1). In the presence of IBTX retigabine also attenuated methoxamine-induced contractions compared to these contractions in the presence of IBTX alone (Figure 3A1). Thus, the anti-contractile effect of retigabine was enhanced by IBTX (Figure 3B1), whereas the contractile effect of IBTX was reduced by retigabine (Figure 3C1). Such effects of IBTX and retigabine were also observed when contractility was assessed based on the myogenic response of gracilis arteries (Supplementary Figures 6A–C).

Retigabine at 10^–5^M attenuated methoxamine-induced contractions, whereas IBTX at 10^–7^M enhanced methoxamine-induced contractions (Figure 3A2). Of note, in the presence of retigabine IBTX did not affect methoxamine-induced contractions compared to these contractions in the presence of retigabine alone (Figure 3A2). In the presence of IBTX retigabine also attenuated methoxamine-induced contractions compared to these contractions in the presence of IBTX alone (Figure 3A2). Thus, the anti-contractile effect of retigabine was considerably enhanced by IBTX (Figure 3B2), whereas the contractile effect of IBTX was abolished by retigabine (Figure 3C2).

#### The Functional Impact of Kv7 Channels Decreases After Activating BK Channels

**FIGURE 4:**
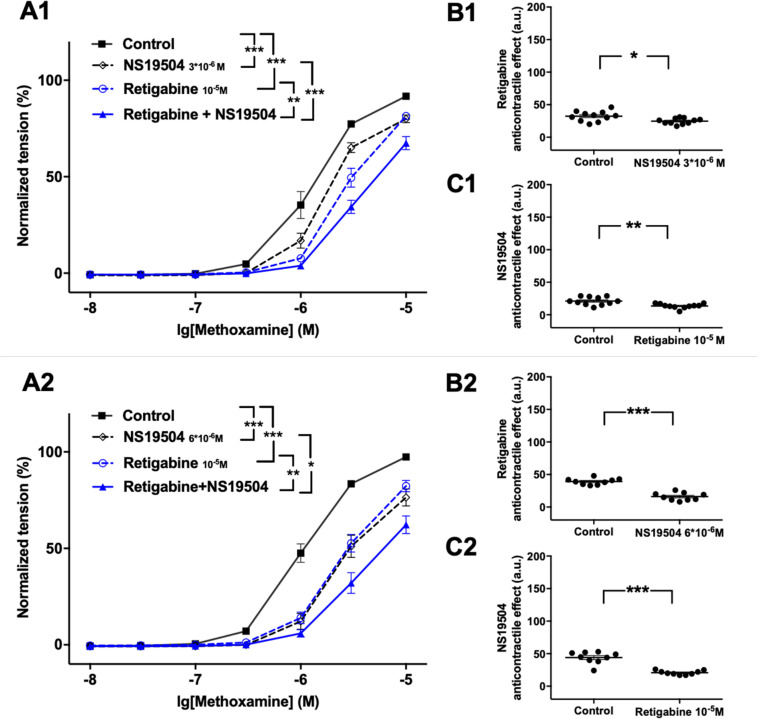
Effect of retigabine and NS19504 on methoxamine-induced contractions of the saphenous artery. **(A1,A2)** Normalized tension of saphenous arteries at different methoxamine concentration in the absence of potassium channel active agents (Control), in the presence of retigabine (Retigabine 10^–5^M), in the presence of NS19504 (**A1**: NS19504 3*10^–6^M, A2: NS19504 6*10^–6^M) and in the combined presence of retigabine and NS19504 (Retigabine + NS19504). **(B1,B2)** Retigabine anti-contractile effect in the absence (Control) and presence of NS19504 (**B1**: NS19504 3*10^–6^M, B2: NS19504 6*10^–6^M). **(C1,C2)** NS19504 anti-contractile effect in the absence (Control) and presence of retigabine (Retigabine 10^–5^M). n1 = 11, n2 = 9; *p < 0.05, **p < 0.01; ***p < 0.001.

Retigabine at 10^–5^M as well as NS19504 at 3^∗^10^–6^M attenuated methoxamine-induced contractions (Figure 4A1). In the presence of NS19504 retigabine also attenuated methoxamine-induced contractions compared to these contractions in the presence of NS19504 alone (Figure 4A1). Further, in the presence of retigabine NS19504 attenuated methoxamine-induced contractions compared to these contractions in the presence of retigabine alone (Figure 4A1). Thus, the anti-contractile effect of retigabine was reduced by NS19504 (Figure 4B1), and the anti-contractile effect of NS19504 was reduced by retigabine (Figure 4C1). The same effects where observed for NS19504 at 6^∗^10^–6^M (Figures 4A2,B2,C2). Such effects of NS19504 and retigabine were also observed when contractility was assessed based on the myogenic response of gracilis arteries (Supplementary Figures 7A–C).

**FIGURE 5:**
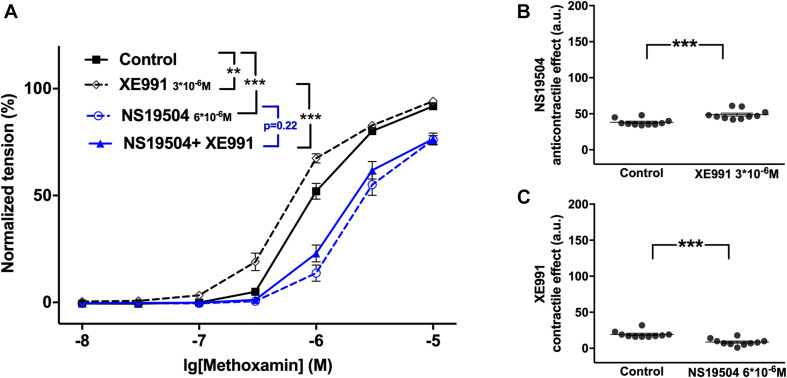
Effect of NS19504 and XE991 on methoxamine-induced contractions of the saphenous artery. **(A)** Normalized tension of saphenous arteries with different methoxamine concentrations in the absence of potassium channel active agents (Control), in the presence of XE991 (XE991 3*10^–6^M), in the presence of NS19504 (NS19504 6*10^–6^M) and in the combined presence of NS19504 and XE991 (NS19504 + XE991). **(B)** NS19504 anti-contractile effect in the absence (Control) and presence of XE991 (XE991 3*10^–6^M). **(C)** XE991 contractile effect in the absence (Control) and presence of NS19504 (NS19504 6*10^–6^M). *n* = 10; **p < 0.01; ***p < 0.001.

Further, NS19504 at 6^∗^10^–6^M attenuated methoxamine-induced contractions, whereas XE991 at 3^∗^10^–6^M enhanced methoxamine-induced contractions (Figure 5A). In the presence of NS19504 XE991 did not affect methoxamine-induced contractions compared to these contractions in the presence of NS19504 alone (Figure 5A). In the presence of XE991 NS19504 also attenuated methoxamine-induced contractions compared to these contractions in the presence of XE991 alone (Figure 5A). Thus, the anti-contractile effect of NS19504 was enhanced by XE991 (Figure 5B), whereas the contractile effect of XE991 was abolished by NS19504 (Figure 5C). Such effects of XE991 and NS19504 were also observed when contractility was assessed based on the myogenic response of gracilis arteries (Supplementary Figures 8A–C).

### The Functional Impact of BK and Kv7 Channels in Arteries of Young Rats

The functional impact of BK and Kv7 channels on methoxamine-induced contractions was determined also in saphenous arteries of young rats.

#### Expression of Kv7 and BK Channel Genes in Saphenous Arteries of Adult and Young Rats

**FIGURE 6:**
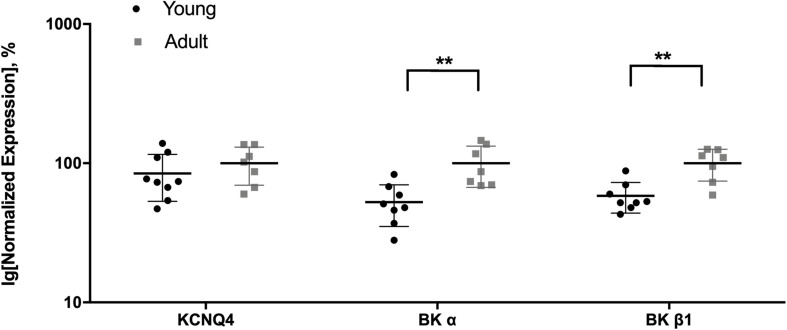
Expression of KCNQ4 and BK channel genes in the saphenous artery of young and adult rats. Normalized expression (related to Gapdh and normalized to the mean of the expression of vessels from adult rats) of KCNQ4 and BK channel genes in saphenous arteries from young (*n* = 8) and adult (*n* = 7) rats. **p < 0.01.

Comparison of the most highly expressed potassium channel genes of interest in the saphenous artery showed that the expression of BKα and BKβ1 was smaller in vessels from young compared to adult rats, whereas for KCNQ4 no difference was detected (Figure 6). Thus, vessels of young rats may serve as a model of a functional situation resembling a considerable block of BK channels.

#### Effect of Retigabine and XE991 on Arterial Contraction

Retigabine at 3^∗^10^–5^M attenuated methoxamine-induced contractions, whereas XE991 at 3^∗^10^–6^M strongly enhanced this effect (Supplementary Figure 9A). In the presence of retigabine XE991 enhanced methoxamine-induced contractions compared to these contractions in the presence of retigabine alone (Supplementary Figure 9A). Of note, in the presence of XE991 retigabine was without effect on methoxamine-induced contractions compared to these contractions in the presence of XE991 alone (Supplementary Figure 9A). Thus, the anti-contractile effect of retigabine was abolished by XE991 (Supplementary Figure 9B).

#### Effect of NS19504 and IBTX on Arterial Contraction

NS19504 at 6^∗^10^–6^M attenuated methoxamine-induced contractions, while IBTX at 10^–7^M enhanced it (Supplementary Figure 10A). In the presence of NS19504, IBTX also enhanced methoxamine-induced contractions compared to these contractions in the presence of NS19504 alone (Supplementary Figure 10A). Of note, in the presence of IBTX NS19504 was without effect on methoxamine-induced contractions compared to these contractions in the presence of IBTX alone (Supplementary Figure 10A). Thus, the anti-contractile effect of NS19504 was abolished by IBTX (Supplementary Figure 10B).

**FIGURE 7:**
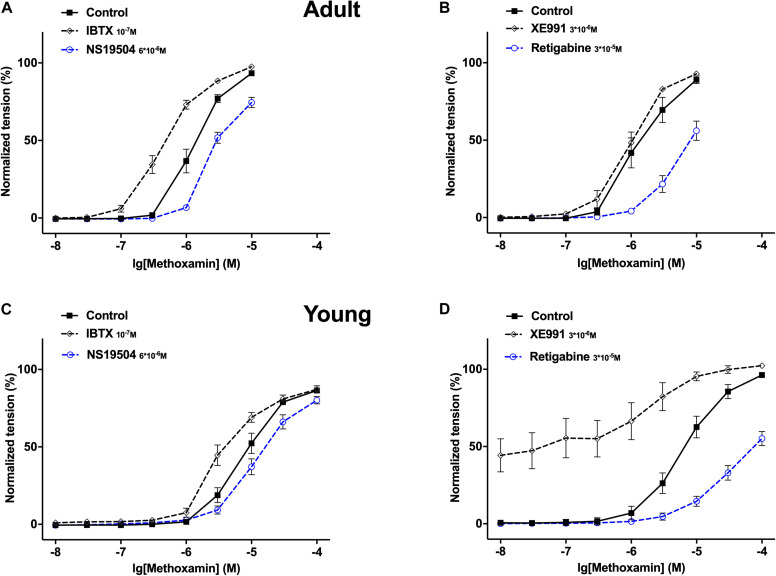
Effect of NS19504, IBTX, Retigabine and XE991 on methoxamine-induced contractions of the saphenous artery in adult and young rats. **(A)** Normalized tension of adult saphenous arteries with different methoxamine concentrations in the absence of BK channel active agents (Control), in the presence of IBTX (IBTX 10^–7^M) and in the presence of NS19504 (NS19504 6*10^–6^M). **(B)** Normalized tension of adult saphenous arteries at different methoxamine concentrations in the absence of Kv7 channel active agents (Control), in the presence of XE991 (XE991 3*10^–6^M), in the presence of retigabine (Retigabine 3*10^–5^M). **(C)** Normalized tension of young saphenous arteries with different methoxamine concentrations in the absence of BK channel active agents (Control), in the presence of IBTX (IBTX 10^–7^M) and in the presence of NS19504 (NS19504 6*10^–6^M). **(D)** Normalized tension of young saphenous arteries at different methoxamine concentrations in the absence of Kv7 channel active agents (Control), in the presence of XE991 (XE991 3*10^–6^M), in the presence of retigabine (Retigabine 3*10^–5^M).

Together these data show that (i) in adults IBTX and NS19504 shifted the concentration-response relationship of methoxamine to the left and to the right, respectively, to a similar degree (Figure 7A) demonstrating that BK channels exert an anticontractile effect during methoxamine-induced contractions; (ii) in young rats, a similar effect of IBTX and NS19504 was observed, albeit with smaller magnitude (Figure 7C) also demonstrating an anticontractile effect of BK channels; (iii) in adults XE991 had no considerable effect on the methoxamine-induced concentration-response relationship, whereas retigabine shifted the concentration-response relationship of methoxamine to the right (Figure 7B), demonstrating that Kv7 channels showed no considerable anticontractile effect; (iv) in contrast in young rats, XE991 and retigabine shifted the concentration-response relationship of methoxamine to the left and to the right, respectively, where the effect of XE991 is more pronounced (Figure 7D) demonstrating a strong anticontractile effect of Kv7 channels.

### The Functional Impact of Kv7 Channels Changed Little After Blocking BK Channels in Young Rats

**FIGURE 8:**
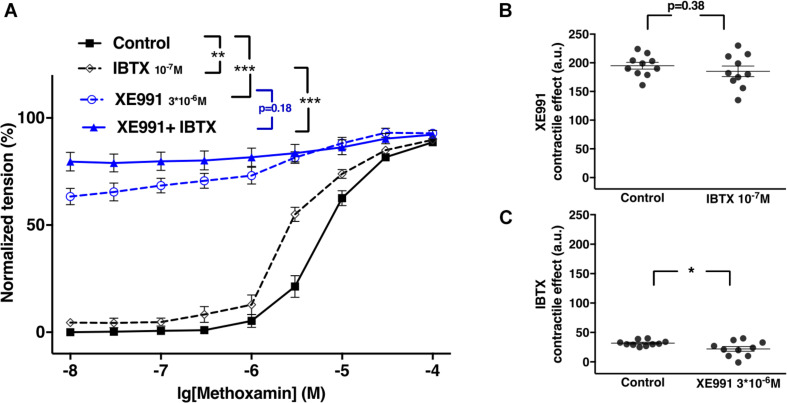
Effect of XE991 and IBTX on methoxamine-induced contractions of the saphenous artery of young rats. **(A)** Normalized tension of saphenous arteries with different methoxamine concentrations in the absence of potassium channel active agents (Control), in the presence of IBTX (IBTX 10^–7^M), in the presence of XE991 (XE991 3*10^–6^M) and in the combined presence of XE991 and IBTX (XE991 + IBTX). **(B)** XE991 contractile effect in the absence (Control) and presence of IBTX (IBTX 10^–7^M). **(C)** IBTX contractile effect in the absence (Control) and presence of XE991 (XE991 3*10^–6^M). *n* = 11; *p < 0.05, **p < 0.01; ***p < 0.001.

XE991 at 3^∗^10^–6^M as well as IBTX at 10^–7^M enhanced methoxamine-induced contractions (Figure 8A). In the presence of IBTX, XE991 also enhanced methoxamine-induced contractions compared to these contractions in the presence of IBTX alone (Figure 8A). Further, in the presence of XE991, IBTX did not affect methoxamine-induced contractions compared to these contractions in the presence of XE991 alone (Figure 8A). Thus, the contractile effect of XE991 was not affected by IBTX (Figure 8B), but the contractile effect of IBTX was abolished by XE991 (Figure 8C).

**FIGURE 9:**
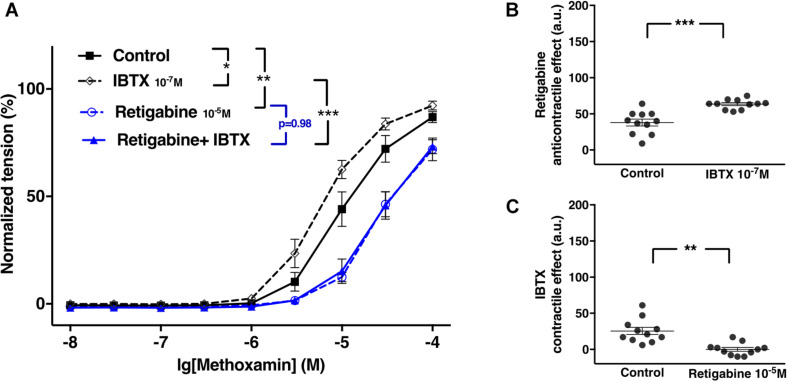
Effect of retigabine and IBTX on methoxamine-induced contractions of the saphenous artery of young rats. **(A)** Normalized tension of saphenous arteries with different methoxamine concentrations in the absence of potassium channel active agents (Control), in the presence of IBTX (IBTX 10^–7^M), in the presence of retigabine (Retigabine 10^–5^M) and in the combined presence of retigabine and IBTX (Retigabine + IBTX). **(B)** Retigabine anti-contractile effect in the absence of (Control) and presence of IBTX (IBTX 10^–7^M). **C)** IBTX contractile effect in the absence of (Control) and presence of retigabine (Retigabine 10^–5^M). *n* = 11; *p < 0.05, **p < 0.01; ***p < 0.001.

Retigabine at 10^–5^M attenuated methoxamine-induced contractions, whereas IBTX at 10^–7^M enhanced methoxamine-induced contractions (Figure 9A). Of note, in the presence of retigabine IBTX did not affect methoxamine-induced contractions compared to these contractions in the presence of retigabine alone (Figure 9A). In the presence of IBTX retigabine also attenuated methoxamine-induced contractions compared to these contractions in the presence of IBTX alone (Figure 9A). Thus, the anti-contractile effect of retigabine was enhanced by IBTX (Figure 9B), whereas the contractile effect of IBTX was abolished by retigabine (Figure 9C).

**FIGURE 10:**
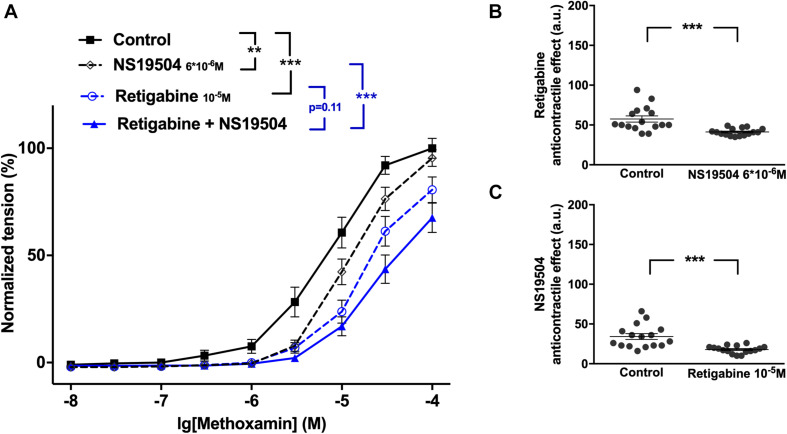
Effect of retigabine and NS19504 on methoxamine-induced contractions of the saphenous artery of young rats. **(A)** Normalized tension of saphenous arteries at different methoxamine concentration in the absence of potassium channel active agents (Control), in the presence of retigabine (Retigabine 10^–5^M), in the presence of NS19504 (NS19504 6^∗^10^–6^M) and in the combined presence of retigabine and NS19504 (Retigabine + NS19504). **(B)** Retigabine anti-contractile effect in the absence (Control) and presence of NS19504 (NS19504 6^∗^10^–6^M). **(C)** NS19504 anti-contractile effect in the absence (Control) and presence of retigabine (Retigabine 10^–5^M). *n* = 16; ^∗∗^*p* < 0.01; ^∗∗∗^*p* < 0.001.

Retigabine at 10^–5^M as well as NS19504 at 6^∗^10^–6^M attenuated methoxamine-induced contractions (Figure 10A). In the presence of NS19504 retigabine also attenuated methoxamine-induced contractions compared to these contractions in the presence of NS19504 alone (Figure 10A). Further, in the presence of retigabine NS19504 did not affect methoxamine-induced contractions compared to these contractions in the presence of retigabine alone (Figure 10A). Thus, the anti-contractile effect of retigabine was reduced by NS19504 (Figure 10B), and the anti-contractile effect of NS19504 was abolished by retigabine (Figure 10C).

**FIGURE 11:**
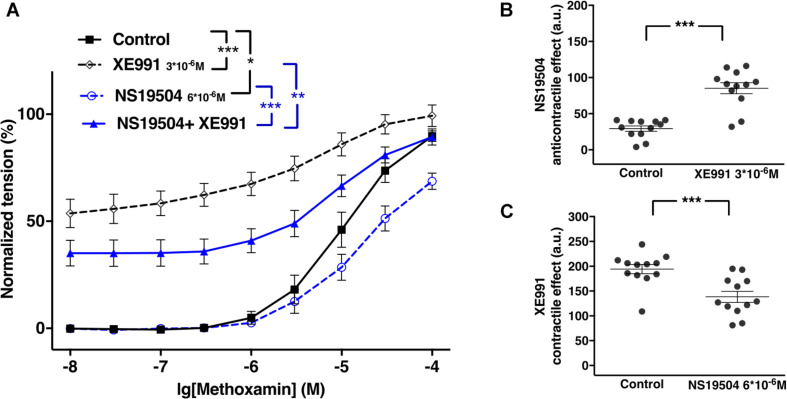
Effect of NS19504 and XE991 on methoxamine-induced contractions of the saphenous artery of young rats. **(A)** Normalized tension of saphenous arteries with different methoxamine concentrations in the absence of potassium channel active agents (Control), in the presence of XE991 (XE991 3*10^–6^M), in the presence of NS19504 (NS19504 6*10^–6^M) and in the combined presence of NS19504 and XE991 (NS19504 + XE991). **(B)** NS19504 anti-contractile effect in the absence (Control) and presence of XE991 (XE991 3*10^–6^M). **(C)** XE991 contractile effect in the absence (Control) and presence of NS19504 (NS19504 6*10^–6^M). *n* = 12; *p < 0.05, **p < 0.01; ***p < 0.001.

NS19504 at 6^∗^10^–6^M attenuated methoxamine-induced contractions, whereas XE991 at 3^∗^10^–6^M enhanced methoxamine-induced contractions (Figure 11A). In the presence of NS19504, XE991 also enhanced methoxamine-induced contractions compared to these contractions in the presence of NS19504 alone (Figure 11A). In the presence of XE991, NS19504 also attenuated methoxamine-induced contractions compared to these contractions in the presence of XE991 alone (Figure 11A). Thus, the anti-contractile effect of NS19504 was enhanced by XE991 (Figure 11B), whereas the contractile effect of XE991 was reduced by NS19504 (Figure 11C).

## Discussion

### Expression of Kv7 and BK Channels in Arteries of Adult Rats

The expression of mRNA for Kv7 (KCNQ) and BK channels was determined in two types of skeletal muscle arteries, the larger saphenous artery and the smaller gracilis artery. The potassium channel genes studied were expressed at different levels. In particular, BK channels (BK α and BK β1 subunits) prevail as in other systemic blood vessels (Nelson and Quayle, 1995; Tykocki et al., 2017). Further, KCNQ4 is the most highly expressed KCNQ gene, followed by KCNQ1 and KCNQ5. This expression profile is similar to other rat (e.g., gracilis arteries, cerebral arteries, coronary arteries, pulmonary arteries and mesenteric arteries) and human arteries (e.g., visceral arteries and mesenteric arteries) (Søgaard et al., 2001; Joshi et al., 2009; Zhong et al., 2010; Ng et al., 2011; Zavaritskaya et al., 2013; Haick and Byron, 2016). The observed expression pattern was similar in both vessels studied. In addition, a comparable expression profile was detected for most channels in endothelium-intact and endothelium-denuded vessels. In contrast, eNOS, expressed preferentially in endothelial cells, showed a much smaller expression in endothelium-denuded vessels. Thus, our findings indicate that the potassium channel genes studied are not predominantly expressed in the endothelium but are expressed in both the endothelium and smooth muscle. An exception is KNCQ4, which was expressed at a lower level in endothelium-intact vessels, pointing to a preferential expression of this channel in smooth muscle cells. To enable the investigation of the functional role of Kv7 and BK channels located in smooth muscle cells, vessels without endothelium have been used in the functional experiments in this study.

### Function of Kv7 and BK Channels in Arteries of Adult Rats

The myogenic response as well as vasoactive substance-induced contractions are two main mechanisms governing vascular contractility (Brozovich et al., 2016). Therefore, in this study, the interaction of Kv7 and BK channels in the regulation of the contractility of isolated arteries was examined based on pressure-induced myogenic responses and methoxamine-induced contractions. The characteristics of the myogenic response of the gracilis artery observed in this study are in accordance with the characteristics of typical myogenic responses (Bayliss, 1902; Davis and Hill, 1999; Schubert et al., 2008). Methoxamine-induced contractions of the saphenous artery have been described previously (Shvetsova et al., 2019).

Of note, in several experiments K channel blocker or their combination produced an increase in basal tone of isometric vessel preparations. Also under these conditions the lowest concentration of methoxamine used (10 nM) did not produce an increase of vessel tension on its own. Thus, the increased values of vessel tension at 10nM methoxamine (most obvious to see in Figures 2, 8, 11 and Supplementary Figure 9) do not represent an increased sensitivity to low concentrations of methoxamine. In fact, they show the increased basal tone induced by K channel blockers or their combinations.

#### Negative Feedback Regulation of Vascular Contractility by Kv7 and BK Channels

The BK channel blocker IBTX enhanced methoxamine-induced contractions and strengthened the myogenic response; the Kv7 channel blocker XE991 also strengthened the myogenic response but did not affect considerably methoxamine-induced contractions in adult animals. NS19504, the BK channel opener, and retigabine, the Kv7 channel opener both attenuated methoxamine-induced contractions and the myogenic response. These data are supported by previous observations in different arteries (Brayden and Nelson, 1992; Rosenfeld et al., 2000; Greenwood and Ohya, 2009; Hill et al., 2010; Zhong et al., 2010; Haick and Byron, 2016; Tykocki et al., 2017; Schmid et al., 2018; Shvetsova et al., 2019). In summary, the data of the present study suggest that Kv7 and BK channels play a negative feedback role in methoxamine-induced contractions as well as in the myogenic response to prevent excessive vasoconstriction by limiting agonist- and pressure-induced depolarization.

The data suggest that Kv7 and BK channels can replace each other, at least regarding the studied negative feedback regulation of methoxamine-induced contraction and the myogenic response. However, this was observed only for the saphenous artery from adult animals. In this vessel from young animals BK channels cannot substitute Kv7 channels. Since BK and Kv7 channels have been shown to differ in their expression and/or functional impact in various vessels, we suggest that in some vessels (like adult saphenous arteries) they can substitute for one another but in other vessels (like young saphenous arteries) either BK or Kv7 channels will secure the negative feedback regulation of vasocontraction. Thus, both channels are needed to ensure negative feedback regulation of vasocontraction in all vessels. The reason for differences in expression and/or functional impact of BK and Kv7 channels in different vessels has to be established yet.

The presented data also show that, at the concentrations used in this study, the Kv7 channel blocker XE991 and the BK channel blocker IBTX are able to abolish the effects of the Kv7 channel opener retigabine and the BK channel opener NS19504, respectively. Thus, when used at appropriate concentrations these agents are selective openers/blockers of their respective channels (see also selection criteria for the BK and Kv7 channel blocker and opener concentrations in the methods section).

Of note, whereas XE991, IBTX and retigabine have been used at the reported concentrations in intact vessels as selective tools to study the function of Kv7 and BK channels previously (Galvez et al., 1990; Wickenden et al., 2000; Yeung and Greenwood, 2005; Ng et al., 2011; Hannigan et al., 2016; Gollasch et al., 2018; Shvetsova et al., 2019; Zavaritskaya et al., 2020), the BK channel opener NS19504 has not been explored on intact vessels before. The data of the present study show, that effects not related to BK channels, i.e., not blocked by IBTX, are observed at higher concentrations of NS19504 (10^–5^ M). However, at lower concentrations (3^∗^10^–6^ M, 6^∗^10^–6^ M) the effects of NS19504 can be blocked completely by IBTX. A similar effect has been observed in guinea pig bladder strips (Nausch et al., 2014). These novel findings demonstrate that at appropriate concentrations NS19504 can be used as a selective opener of vascular smooth muscle BK channels.

#### Regulation of the Functional Impact of Kv7 Channels by BK Channels

Experiments on both pressure-induced myogenic responses and methoxamine-induced contractions in adult rats showed that blockade of BK channels induced or increased a contractile effect of the Kv7 channel blocker XE991 and increased the anti-contractile effect of the Kv7 channel opener retigabine. This suggests that the functional impact of Kv7 channels increases after blocking BK channels. Accordingly, the opposite action on BK channels, their activation, decreased the anti-contractile effect of the Kv7 channel opener retigabine and abolished the contractile effect of the Kv7 channel blocker XE991. These data show that inhibition of BK channels increased the functional impact of Kv7 channels, while activation of BK channels decreased the functional impact of Kv7 channels.

Our findings can be explained based on a recently published idea (Coleman et al., 2017). When functional BK channels have been inhibited by IBTX the accompanying depolarization will move the membrane potential away from the potassium equilibrium potential resulting in a larger driving force (electrochemical gradient) for potassium ions. Further, Kv7 and BK channels are part of a plasma membrane equivalent electrical circuit where each type of potassium channel is in parallel contributing to the total membrane resistance. Blocking BK channels will increase membrane resistance so that for a given change in potassium current a larger change in membrane potential is achieved. In summary, due to the larger driving force and the increased membrane resistance after blocking BK channels changes in Kv7 channel activity, induced by either blockers or openers, will result in a larger change in membrane potential and vessel tension. Furthermore, when functional BK channels have been stimulated by NS19504 the accompanying hyperpolarization will move the membrane potential closer to the potassium equilibrium potential resulting in a smaller driving force for potassium ions. Further, activating BK channels will decrease membrane resistance so that for a given change in potassium current a smaller change in membrane potential is achieved. In summary, due to the smaller driving force and the decreased membrane resistance after activating BK channels, changes in Kv7 channel activity, induced by either blockers or openers, will result in a smaller change in membrane potential and vessel tension. Of note, this reasoning has been successfully applied to explain the differential effects of dual Kv7 and BK channel opener substances, GoSlo compounds (Zavaritskaya et al., 2020).

Of note, a detailed electrophysiological examination (either membrane potential or ion currents) of this model would help to clarify this idea further. The lack of such data is a weakness of the present study. However, this complex and extensive exploration is beyond the scope of the present study.

Notwithstanding the focus of the present study, the Kv7 channels, these data also show that inhibition of Kv7 channels increased the functional impact of BK channels, while activation of Kv7 channels decreased the functional impact of BK channels. Thus, changes in the activity of Kv7 channels affect the activity of BK channels, and vice versa.

#### The Functional Impact of Kv7 Channels Is Suppressed Considerably by BK Channels in Adult but Not in Young Rats

As IBTX, XE991, NS19504 and retigabine, when used at appropriate concentrations, are selective blockers/openers of their respective channels, it can be concluded that BK channels exert an anticontractile effect during methoxamine-induced contractions in adult as well as in young rats (see Figures 7A,C). In young rats, however, the degree of modulation of methoxamine-induced contractions was somewhat smaller compared to adult rats. In contrast, Kv7 channels showed a very small anticontractile effect in adult rats, but a strong anticontractile effect in young rats (see Figures 7B,D). These data are consistent with and nicely reproduce recently published findings showing a strongly increased negative feedback regulation of vasocontraction by Kv7 channels in arteries of young rats (compared to adult animals) which was accompanied by a lower BK channel gene expression and an increased abundance of Kv7 channels (Figure 6 and Shvetsova et al., 2019).

Comparative evaluation of the functional impact of Kv7 and BK channels on arterial contractility in adult and young rats was performed on the basis of methoxamine-induced contractions. Pressure-induced myogenic responses could not be employed because of the very small size of the gracilis artery in young rats. As described above in detail, in adult rats, inhibition of BK channels increased the functional impact of Kv7 channels, while activation of BK channels decreased the functional impact of Kv7 channels. Interestingly, in young rats, blockade of BK channels did not affect the contractile effect of the Kv7 channel blocker XE991 (in contrast to adult rats) but increased the anti-contractile effect of the Kv7 channel opener retigabine (as in adult rats). The opposite action on BK channels, their activation, decreased the anti-contractile effect of the Kv7 channel opener retigabine (as in adult rats) and reduced the contractile effect of the Kv7 channel blocker XE991 (as in adult rats).

**FIGURE 12:**
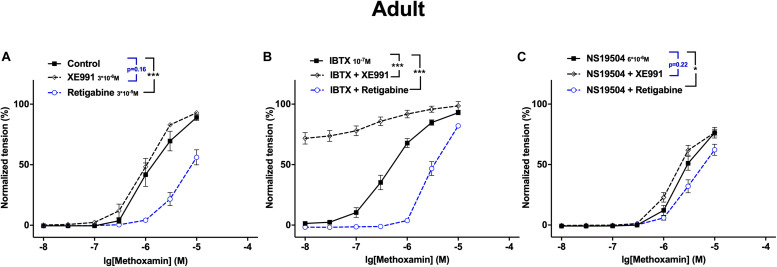
Effect of Retigabine, XE991, NS19504 and IBTX on methoxamine-induced contractions of the saphenous artery of adult rats. **(A)** Normalized tension of saphenous arteries at different methoxamine concentrations in the absence of Kv7 channel active agents (Control), in the presence of XE991 (XE991 3*10^–6^M), in the presence of retigabine (Retigabine 3*10^–5^M). **(B)** Normalized tension of saphenous arteries with different methoxamine concentrations in the presence of IBTX (IBTX 10^–7^M), in the combined presence of IBTX and XE991 (IBTX + XE991), and in the combined presence of IBTX and retigabine (IBTX + Retigabine). **(C)** Normalized tension of saphenous arteries with different methoxamine concentrations in the presence of NS19504 (NS19504 6*10^–6^M), in the combined presence of NS19504 and XE991 (NS19504 + XE991) and in the combined presence of NS19504 and retigabine (NS19504 + Retigabine). *p < 0.05, ***p < 0.001.

**FIGURE 13:**
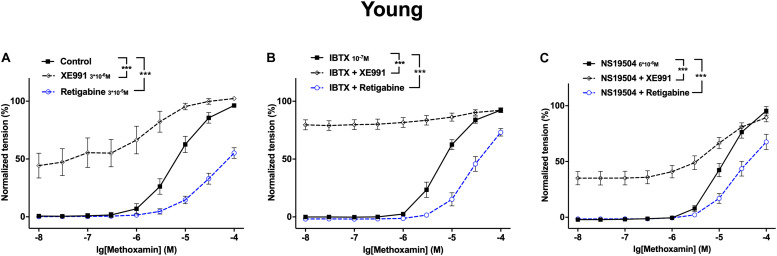
Effect of Retigabine, XE991, NS19504 and IBTX on methoxamine-induced contractions of the saphenous artery of young rats. **(A)** Normalized tension of saphenous arteries at different methoxamine concentrations in the absence of Kv7 channel active agents (Control), in the presence of XE991 (XE991 3*10^–6^M), in the presence of retigabine (Retigabine 3*10^–5^M). **(B)** Normalized tension of saphenous arteries with different methoxamine concentrations in the presence of IBTX (IBTX 10^–7^M), in the combined presence of IBTX and XE991 (IBTX + XE991), and in the combined presence of IBTX and retigabine (IBTX + Retigabine). **(C)** Normalized tension of saphenous arteries with different methoxamine concentrations in the presence of NS19504 (NS19504 6*10^–6^M), in the combined presence of NS19504 and XE991 (NS19504 + XE991) and in the combined presence of NS19504 and retigabine (NS19504 + Retigabine). ***p < 0.001.

A different look at the data revealed that in adult rats Kv7 channels showed no considerable anticontractile effect on methoxamine-induced contraction, although they are available as the effect of retigabine shows (Figure 12A). However, a considerable anticontractile effect of Kv7 channels was observed after blockade of BK channels (Figure 12B); this effect was eliminated after activation of BK channels (Figure 12C). Thus, the functional impact of Kv7 channels is limited by BK channels in adult rats. In young rats, Kv7 channels showed a strong anticontractile effect on methoxamine-induced contraction (Figure 13A). This effect was still observed after blockade (Figure 13B) as well as after activation (Figure 13C) of BK channels. Only the range of vessel tension that Kv7 channels could affect was increased after blockade and decreased after activation of BK channels (Figures 13B,C). Thus, the functional impact of Kv7 channels is not limited by BK channels in young rats.

This observation is in accordance with the idea mentioned above (Coleman et al., 2017). In young rats BK channels exerted an anticontractile effect during methoxamine-induced contractions, as in adult rats. However, in young rats, the range of vessel tension that BK channels could affect was smaller. Thus, vessels of young rats were in a functional situation resembling a considerable block of BK channels. When the remaining functional BK channels have been blocked by IBTX the accompanying depolarization will be small, and will almost not move the membrane potential in relation to the potassium equilibrium potential resulting in only a slightly larger driving force for potassium ions. Further, blocking BK channels will almost not increase membrane resistance so that for a given change in potassium current only a slightly increased change in membrane potential is achieved. In summary, due to the very small increase in driving force and membrane resistance after blocking BK channels, blockade of Kv7 channels will result in almost the same change in membrane potential and vessel tension as with unblocked BK channels. Furthermore, when functional BK channels have been stimulated by NS19504 the accompanying hyperpolarization will move the membrane potential closer to the potassium equilibrium potential resulting in a smaller driving force for potassium ions. Further, BK channels activation will decrease membrane resistance so that for a given change in potassium current a smaller change in membrane potential is achieved. In summary, due to the smaller driving force and the decreased membrane resistance after activation of BK channels changes in Kv7 channel activity, induced by either blockers or openers, will result in a smaller change in membrane potential and vessel tension.

## Conclusion

**FIGURE 14:**
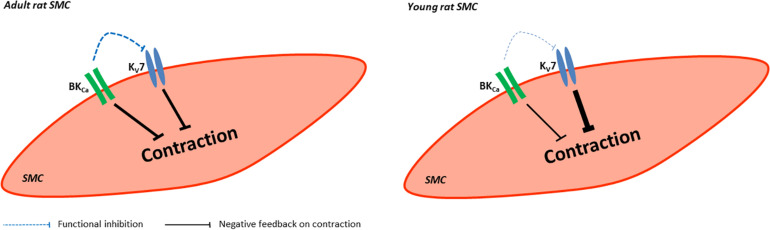
Summary of the role of BK and KV7 channels in saphenous arteries of young and adult rats. In vessels from adult rats BK and Kv7 channels evoke a similar negative feedback regulation on vessel contraction whereby BK channels produce a considerable functional inhibition on Kv7 channels. In vessels from young rats BK and Kv7 channels evoke different negative feedback regulation on vessel contraction with Kv7 channels dominating. BK channels produce almost no functional inhibition on Kv7 channels.

Kv7 channels and BK channels are expressed in rat arteries, the saphenous and gracilis arteries. They mainly function as a negative feedback mechanism in the regulation of the contractility of these arteries. In adult rats, the functional impact of Kv7 channels is suppressed by BK channels, this effect was not observed in young rats (Figure 14).

As an additional finding, it was observed that the selectivity of NS19504 for BK channels is concentration dependent. Under the conditions of this study, NS19504 at 6^∗^10^–6^ M selectively activates vascular smooth muscle BK channels.

## Data Availability Statement

The raw data supporting the conclusions of this article will be made available by the authors, without undue reservation.

## Ethics Statement

The study was reviewed and approved by Regierungspräsidium Karlsruhe, Germany (I-17/17).

## Author Contributions

DM and RS designed the study and wrote the manuscript. DM, DG, MM, and NS performed the experiments. DM, DG, MM, NS, and RS analyzed the data and read and approved the manuscript. All the authors contributed to the article and approved the submitted version.

## Conflict of Interest

The authors declare that the research was conducted in the absence of any commercial or financial relationships that could be construed as a potential conflict of interest.

## Footnotes

1 www.bio-rad.com

## Supplementary Material

Supplementary Figure 1 Effect of retigabine and XE991 on methoxamine-induced contractions of the saphenous artery. **(A)** Original recordings of methoxamine-induced contractions in the presence of XE991 and retigabine in a wire myograph experiment on saphenous arteries. **(B)** Normalized tension of saphenous arteries at different methoxamine concentrations in the absence of Kv7 channel active agents (Control), in the presence of XE991 (XE991 3^∗^10^–6^M), in the presence of retigabine (Retigabine 3^∗^10^–5^M) and in the combined presence of retigabine and XE991 (Retigabine + XE991). **(C)** Anti-contractile effect of retigabine in the absence (Control) and presence of XE991 (XE991 3^∗^10^–6^M). *n* = 8; ^∗∗∗^*p* < 0.001.

Supplementary Figure 2 Effect of retigabine and XE991 on the myogenic response of the gracilis artery. **(A)** Original recordings of myogenic responses in the presence of XE991 and retigabine in a pressure myograph experiment on gracilis arteries**. (B)** Normalized diameter of gracilis arteries at different intra-luminal pressures in the absence of Kv7 channel active agents (Control), in the presence of XE991 (XE991 3^∗^10^–6^M), in the presence of retigabine (Retigabine 3^∗^10^–6^M) and in the combined presence of retigabine and XE991 (Retigabine and XE991). **(C)** Anti-contractile effect of retigabine in the absence (Control) and presence of XE991 (XE991 3^∗^10^–6^M). *n* = 10; ^∗^*p* < 0.05, ^∗∗^*p* < 0.01; ^∗∗∗^*p* < 0.001.

Supplementary Figure 3 Effect of NS19504 and IBTX on methoxamine-induced contractions of the saphenous artery. **(A–D)** Original recordings of methoxamine-induced contractions in the presence of IBTX and NS19504 in a wire myograph experiment on saphenous arteries**. (E1–E3)** Normalized tension of saphenous arteries with different methoxamine concentrations in the absence of BK channel active agents (Control), in the presence of IBTX (IBTX 10^–7^M), in the presence of NS19504 (**E1**: NS19504 3^∗^10^–6^M; **E2**: NS19504 6^∗^10^–6^M; **E3**: NS19504 10^–5^M) and in the combined presence of NS19504 and IBTX (NS19504 and IBTX). **(F1–F3)** NS19504 anti-contractile effect in the absence (Control) and presence of IBTX (IBTX 10^–7^M). n1 = 7; n2 = 7; n3 = 8; ^∗^*p* < 0.05, ^∗∗^*p* < 0.01; ^∗∗∗^*p* < 0.001.

Supplementary Figure 4 Effect of NS19504 and IBTX on the myogenic response of the gracilis artery. **(A)** Original recordings of myogenic responses in the presence of IBTX and NS19504 in a pressure myograph experiment on gracilis arteries. **(B)** Normalized diameter of gracilis arteries at different intra-luminal pressures in the absence of BK channel active agents (Control), in the presence of IBTX (IBTX 10^–7^M), in the presence of NS19504 (NS19504 3^∗^10^–6^M) and in the combined presence of NS19504 and IBTX (NS19504 and IBTX). **(C)** NS19504 anti-contractile effect in the absence (Control) and presence of IBTX (IBTX 10^–7^M). *n* = 10; ^∗^*p* < 0.05, ^∗∗^*p* < 0.01; ^∗∗∗^*p* < 0.001.

Supplementary Figure 5 Effect of XE991 and IBTX on the myogenic response of the gracilis artery. **(A1,A2)** Normalized diameter of gracilis arteries with different intra-luminal pressures in the absence of potassium channel blockers (Control), in the presence of IBTX (IBTX 10^–7^M), in the presence of XE991 (XE991 3^∗^10^–6^M) and in the combined presence of IBTX and XE991 (**A1** in sequence XE991 + IBTX, **A2** in sequence IBTX + XE991). **(B1,B2)** XE991 contractile effect in the absence (Control) and presence of IBTX (IBTX 10^–7^M). **(C1,C2)** IBTX contractile effect in the absence (Control) and presence of XE991 (XE991 3^∗^10^–6^M). n1 = 10, n2 = 10; ^∗^*p* < 0.05, ^∗∗∗^*p* < 0.001.

Supplementary Figure 6 Effect of retigabine and IBTX on the myogenic response of the gracilis artery. **(A)** Normalized diameter of gracilis arteries at different intra-luminal pressures in the absence of potassium channel active agents (Control), in the presence of IBTX (IBTX 10^–7^M), in the presence of retigabine (Retigabine 3^∗^10^–6^M) and in the combined presence of retigabine and IBTX (Retigabine and IBTX). **(B)** Retigabine anti-contractile effect in the absence (Control) and presence of IBTX (IBTX 10^–7^M). **(C)** IBTX contractile effect in the absence (Control) and presence of retigabine (Retigabine 3^∗^10^–6^M). *n* = 10; ^∗^*p* < 0.05, ^∗∗^*p* < 0.01.

Supplementary Figure 7 Effect of retigabine and NS19504 on the myogenic response of the gracilis artery. **(A)** Normalized diameter of gracilis arteries at different intra-luminal pressures in the absence of potassium channel active agents (Control), in the presence of retigabine (Retigabine 3^∗^10^–6^M), in the presence of NS19504 (NS19504 3^∗^10^–6^M) and in the combined presence of retigabine and NS19504 (Retigabine + NS19504). **(B)** Retigabine anti-contractile effect in the absence of (Control) and presence of NS19504 (NS19504 3^∗^10^–6^M). **(C)** NS19504 anti-contractile effect in the absence of (Control) and presence of retigabine (Retigabine 3^∗^10^–6^M). *n* = 9; ^∗^*p* < 0.05, ^∗∗^*p* < 0.01.

Supplementary Figure 8 Effect of NS19504 and XE991 on the myogenic response of the gracilis artery. **(A)** Normalized diameter of gracilis arteries at different intra-luminal pressures in the absence of potassium channel active agents (Control), in the presence of XE991 (XE991 3^∗^10^–6^M), in the presence of NS19504 (NS19504 3^∗^10^–6^M) and in the combined presence of NS19504 and XE991 (NS19504 + XE991). **(B)** NS19504 anti-contractile effect in the absence (Control) and presence of XE991 (XE991 3^∗^10^–6^M). **(C)** XE991 contractile effect in the absence (Control) and presence of NS19504 (NS19504 3^∗^10^–6^M). *n* = 9; ^∗^*p* < 0.05, ^∗∗^*p* < 0.01; ^∗∗∗^*p* < 0.001.

Supplementary Figure 9 Effect of retigabine and XE991 on methoxamine-induced contractions of the saphenous artery of young rats. **(A)** Normalized tension of saphenous arteries at different methoxamine concentrations in the absence of Kv7 channel active agents (Control), in the presence of XE991 (XE991 3^∗^10^–6^M), in the presence of retigabine (Retigabine 3^∗^10^–5^M) and in the combined presence of retigabine and XE991 (Retigabine + XE991). **(B)** Anti-contractile effect of retigabine in the absence (Control) and presence of XE991 (XE991 3^∗^10^–6^M). *n* = 10; ^∗∗∗^*p* < 0.001.

Supplementary Figure 10 Effect of NS19504 and IBTX on methoxamine-induced contractions of the saphenous artery of young rats. **(A)** Normalized tension of saphenous arteries with different methoxamine concentrations in the absence of BK channel active agents (Control), in the presence of IBTX (IBTX 10^–7^M), in the presence of NS19504 (NS19504 6^∗^10^–6^M) and in the combined presence of NS19504 and IBTX (NS19504 and IBTX). **(B)** NS19504 anti-contractile effect in the absence (Control) and presence of IBTX (IBTX 10^–7^M). *n* = 13; ^∗^*p* < 0.05, ^∗∗^*p* < 0.01; ^∗∗∗^*p* < 0.001.
