# Supplementary material for: The Functional Availability of Arterial Kv7 Channels Is Suppressed Considerably by Large-Conductance Calcium-Activated Potassium Channels in 2- to 3-Month Old but Not in 10- to 15-Day Old Rats
Source: Front Physiol. 2020 Dec 15;11:597395. doi: 10.3389/fphys.2020.597395 (PMC7770149; doi:10.3389/fphys.2020.597395)
Supplement: Supplementary file 1 [file Data_Sheet_1.docx]

**Supplement**

Retigabine at 3*10^-6^M attenuated the myogenic response, while XE991 at 3*10^-6^M strengthened it (Fig. S2A, B). In the presence of retigabine XE991 also strengthened the myogenic response compared to this response in the presence of retigabine alone (Fig. S2B). Of note, in the presence of XE991 retigabine was without effect on the myogenic response compared to this response in the presence of XE991 alone (Fig. S2B). Thus, the anti-contractile effect of retigabine was abolished by XE991 (Fig. S2C).

NS19504 at 3*10^-6^M attenuated the myogenic response, while IBTX at 10^-7^M strengthened it (Fig. S4A, B). In the presence of NS19504 IBTX also strengthened the myogenic response compared to this response in the presence of NS19504 alone (Fig. S4B). Of note, in the presence of IBTX NS19504 was without effect on the myogenic response compared to this response in the presence of IBTX alone (Fig. S4B). Thus, the anti-contractile effect of NS19504 was abolished by IBTX (Fig. S4C).

XE991 at 3*10^-6^M as well as IBTX at 10^-7^M strengthened the myogenic response (Fig. S5A1). In the presence of IBTX XE991 also strengthened the myogenic response compared to this response in the presence of IBTX alone (Fig. S5A1). Further, in the presence of XE991 IBTX strengthened the myogenic response compared to these responses in the presence of XE991 alone (Fig. S5A1). Thus, the contractile effect of XE991 was enhanced by IBTX (Fig. S5B1), and the contractile effect of IBTX was enhanced by XE991 (Fig. S5C1). These data were obtained when IBTX was applied first and XE991 was added subsequently. The same results were observed when XE991 was applied first and IBTX was added afterwards (Fig. S5A2, B2, C2).

Retigabine at 3*10^-6^M attenuated the myogenic response, while IBTX at 10^-7^M strengthened it (Fig. S6A). Of note, in the presence of retigabine IBTX did not affect the myogenic response compared to this response in the presence of retigabine alone (Fig. S6A). In the presence of IBTX retigabine also attenuated the myogenic response compared to this response in the presence of IBTX alone (Fig. S6A). Thus, the anti-contractile effect of retigabine was enhanced by IBTX (Fig. S6B), whereas the contractile effect of IBTX was abolished by retigabine (Fig. S6C).

Retigabine at 3*10^-6^M as well as NS19504 at 3*10^-6^M attenuated the myogenic response (Fig. S7A). In the presence of NS19504 retigabine did not affect the myogenic response compared to this response in the presence of NS19504 alone (Fig. S7A). Further, in the presence of retigabine NS19504 did not affect the myogenic response compared to this response in the presence of retigabine alone (Fig. S7A). Thus, the anti-contractile effect of retigabine was abolished by NS19504 (Fig. S7B), and the anti-contractile effect of NS19504 was abolished by retigabine (Fig. S7C).

NS19504 at 3*10^-6^M attenuated the myogenic response, while XE991 at 3*10^-6^M strengthened it (Fig. S8A). Of note, in the presence of NS19504 XE991 did not affect the myogenic response compared to this response in the presence of NS19504 alone (Fig. S8A). In the presence of XE991 NS19504 attenuated the myogenic response compared to this response in the presence of XE991 alone (Fig. S8A). Thus, the anti-contractile effect of NS19504 was enhanced by XE991 (Fig. S8B), whereas the contractile effect of XE991 was abolished by NS19504 (Fig. S8C).
