# Supplementary figures and images for: The Functional Availability of Arterial Kv7 Channels Is Suppressed Considerably by Large-Conductance Calcium-Activated Potassium Channels in 2- to 3-Month Old but Not in 10- to 15-Day Old Rats
Source: Front Physiol. 2020 Dec 15;11:597395. doi: 10.3389/fphys.2020.597395 (PMC7770149; doi:10.3389/fphys.2020.597395)

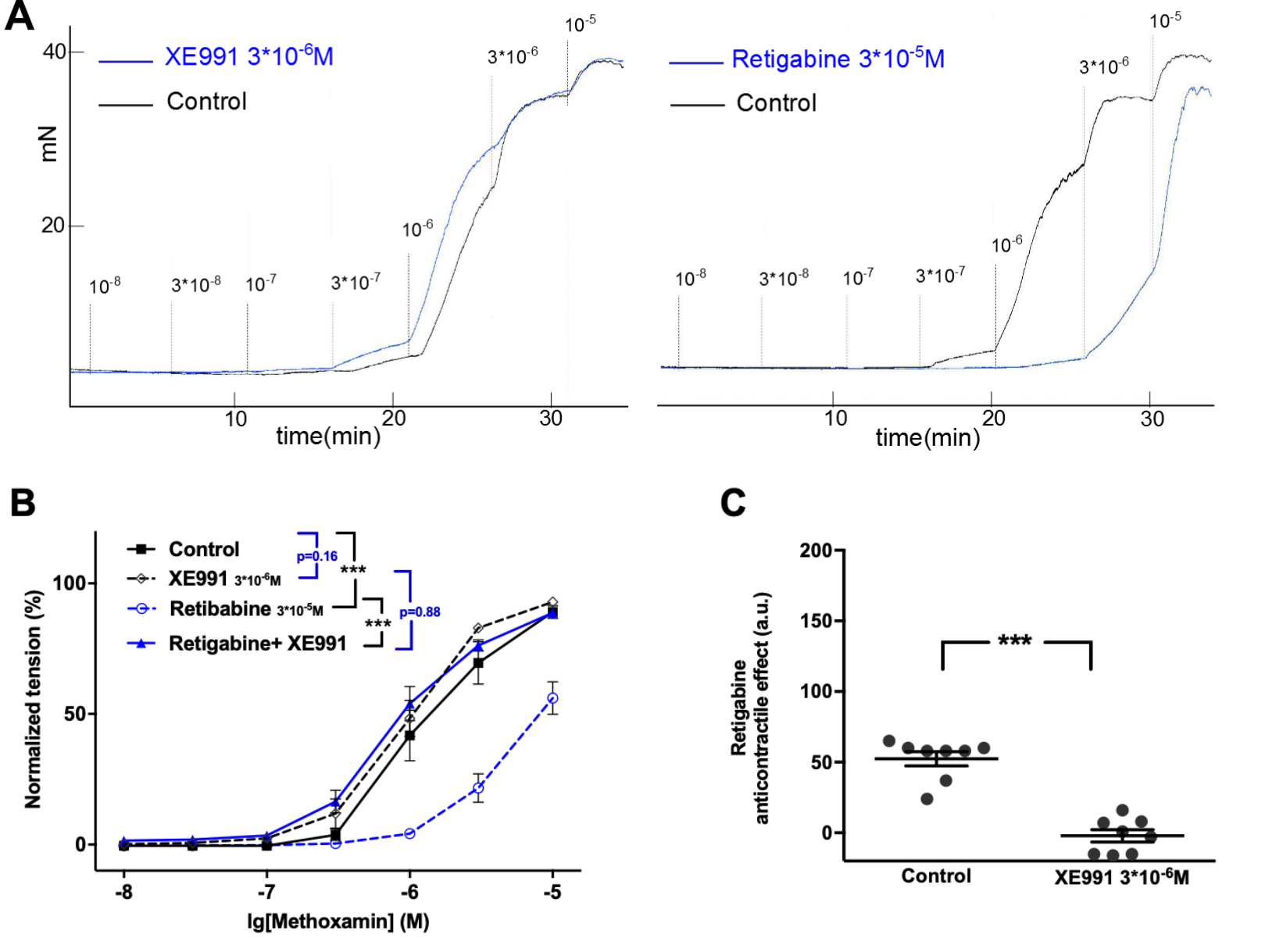

Supplement: Supplementary Figure 1 — Effect of retigabine and XE991 on methoxamine-induced contractions of the saphenous artery. (A) Original recordings of methoxamine-induced contractions in the presence of XE991 and retigabine in a wire myograph experiment on saphenous arteries. (B) Normalized tension of saphenous arteries at different methoxamine concentrations in the absence of Kv7 channel active agents (Control), in the presence of XE991 (XE991 3∗10–6M), in the presence of retigabine (Retigabine 3∗10–5M) and in the combined presence of retigabine and XE991 (Retigabine + XE991). (C) Anti-contractile effect of retigabine in the absence (Control) and presence of XE991 (XE991 3∗10–6M). n = 8; ∗∗∗p < 0.001. [file Image_1.TIFF]

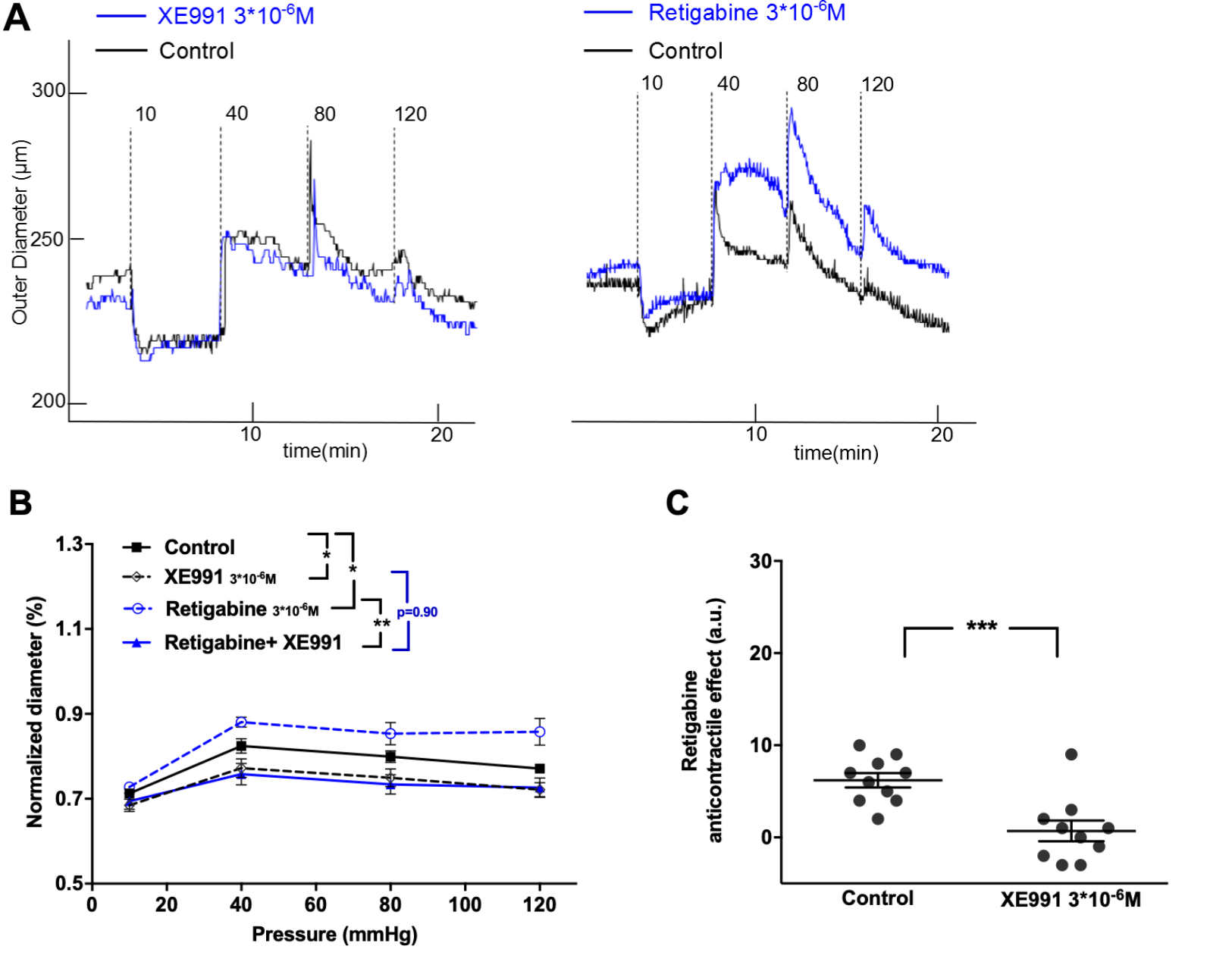

Supplement: Supplementary Figure 2 — Effect of retigabine and XE991 on the myogenic response of the gracilis artery. (A) Original recordings of myogenic responses in the presence of XE991 and retigabine in a pressure myograph experiment on gracilis arteries. (B) Normalized diameter of gracilis arteries at different intra-luminal pressures in the absence of Kv7 channel active agents (Control), in the presence of XE991 (XE991 3∗10–6M), in the presence of retigabine (Retigabine 3∗10–6M) and in the combined presence of retigabine and XE991 (Retigabine and XE991). (C) Anti-contractile effect of retigabine in the absence (Control) and presence of XE991 (XE991 3∗10–6M). n = 10; ∗p < 0.05, ∗∗p < 0.01; ∗∗∗p < 0.001. [file Image_2.TIFF]

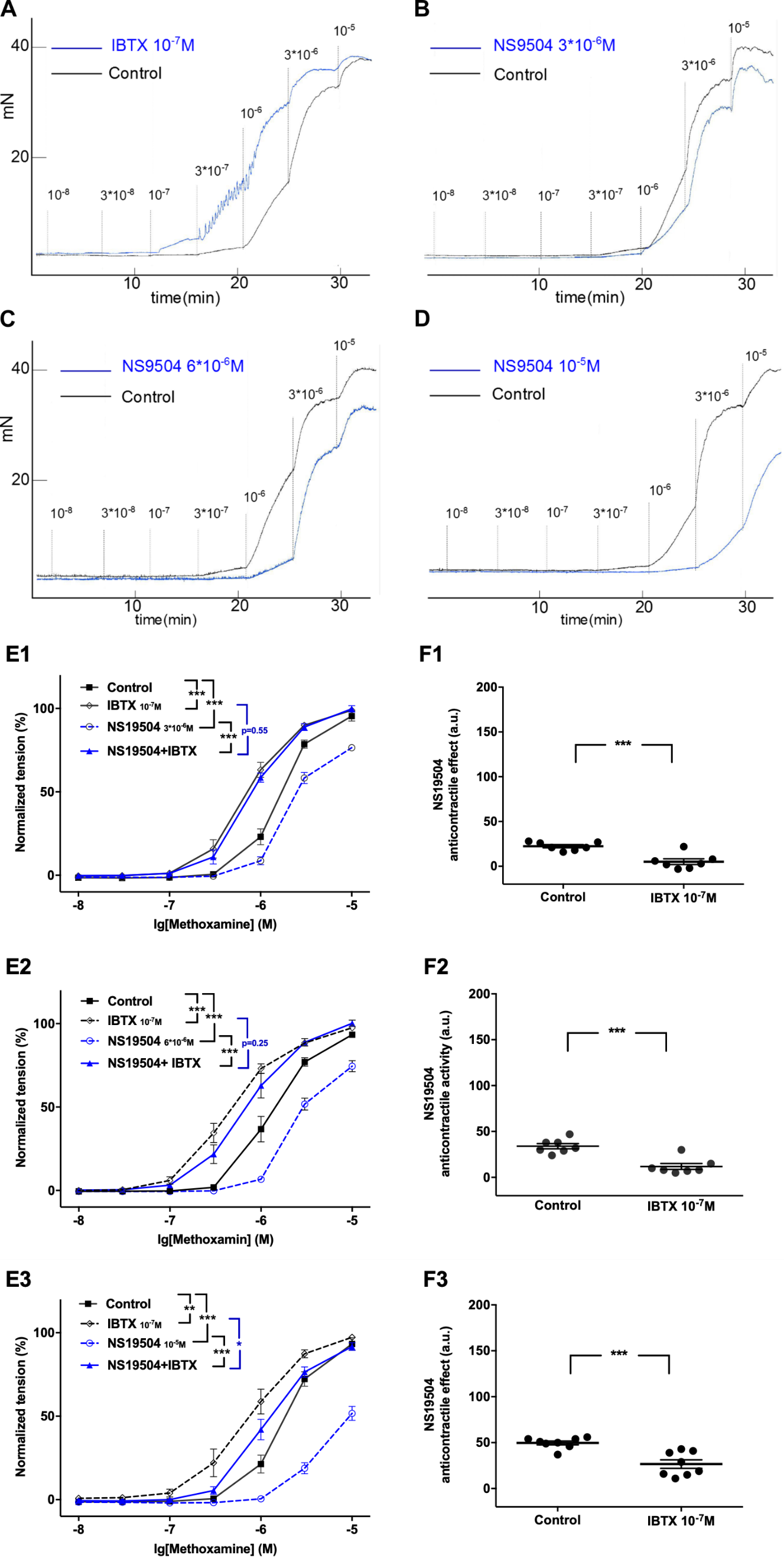

Supplement: Supplementary Figure 3 — Effect of NS19504 and IBTX on methoxamine-induced contractions of the saphenous artery. (A–D) Original recordings of methoxamine-induced contractions in the presence of IBTX and NS19504 in a wire myograph experiment on saphenous arteries. (E1–E3) Normalized tension of saphenous arteries with different methoxamine concentrations in the absence of BK channel active agents (Control), in the presence of IBTX (IBTX 10–7M), in the presence of NS19504 (E1: NS19504 3∗10–6M; E2: NS19504 6∗10–6M; E3: NS19504 10–5M) and in the combined presence of NS19504 and IBTX (NS19504 and IBTX). (F1–F3) NS19504 anti-contractile effect in the absence (Control) and presence of IBTX (IBTX 10–7M). n1 = 7; n2 = 7; n3 = 8; ∗p < 0.05, ∗∗p < 0.01; ∗∗∗p < 0.001. [file Image_3.TIFF]

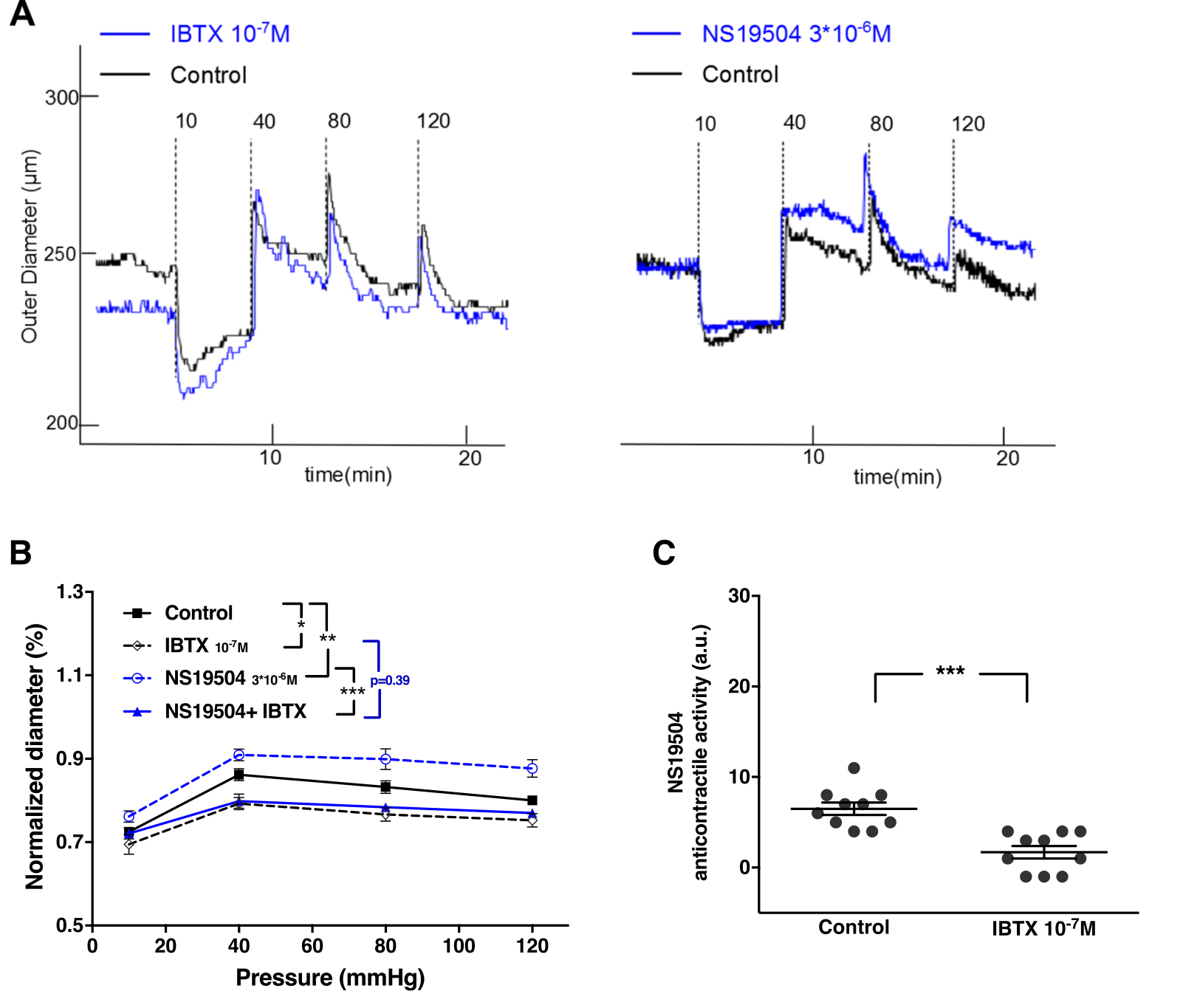

Supplement: Supplementary Figure 4 — Effect of NS19504 and IBTX on the myogenic response of the gracilis artery. (A) Original recordings of myogenic responses in the presence of IBTX and NS19504 in a pressure myograph experiment on gracilis arteries. (B) Normalized diameter of gracilis arteries at different intra-luminal pressures in the absence of BK channel active agents (Control), in the presence of IBTX (IBTX 10–7M), in the presence of NS19504 (NS19504 3∗10–6M) and in the combined presence of NS19504 and IBTX (NS19504 and IBTX). (C) NS19504 anti-contractile effect in the absence (Control) and presence of IBTX (IBTX 10–7M). n = 10; ∗p < 0.05, ∗∗p < 0.01; ∗∗∗p < 0.001. [file Image_4.TIFF]

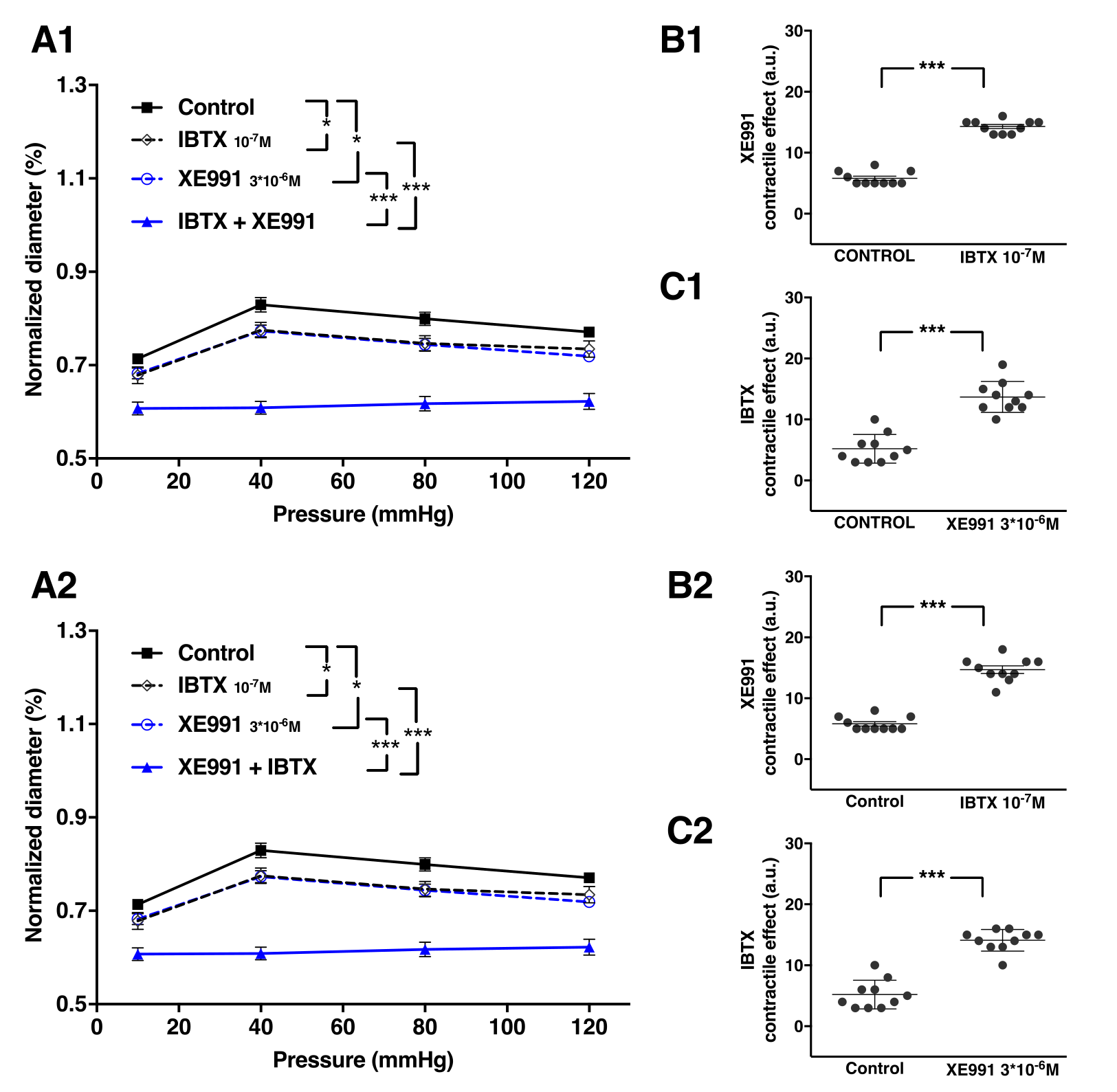

Supplement: Supplementary Figure 5 — Effect of XE991 and IBTX on the myogenic response of the gracilis artery. (A1,A2) Normalized diameter of gracilis arteries with different intra-luminal pressures in the absence of potassium channel blockers (Control), in the presence of IBTX (IBTX 10–7M), in the presence of XE991 (XE991 3∗10–6M) and in the combined presence of IBTX and XE991 (A1 in sequence XE991 + IBTX, A2 in sequence IBTX + XE991). (B1,B2) XE991 contractile effect in the absence (Control) and presence of IBTX (IBTX 10–7M). (C1,C2) IBTX contractile effect in the absence (Control) and presence of XE991 (XE991 3∗10–6M). n1 = 10, n2 = 10; ∗p < 0.05, ∗∗∗p < 0.001. [file Image_5.TIFF]

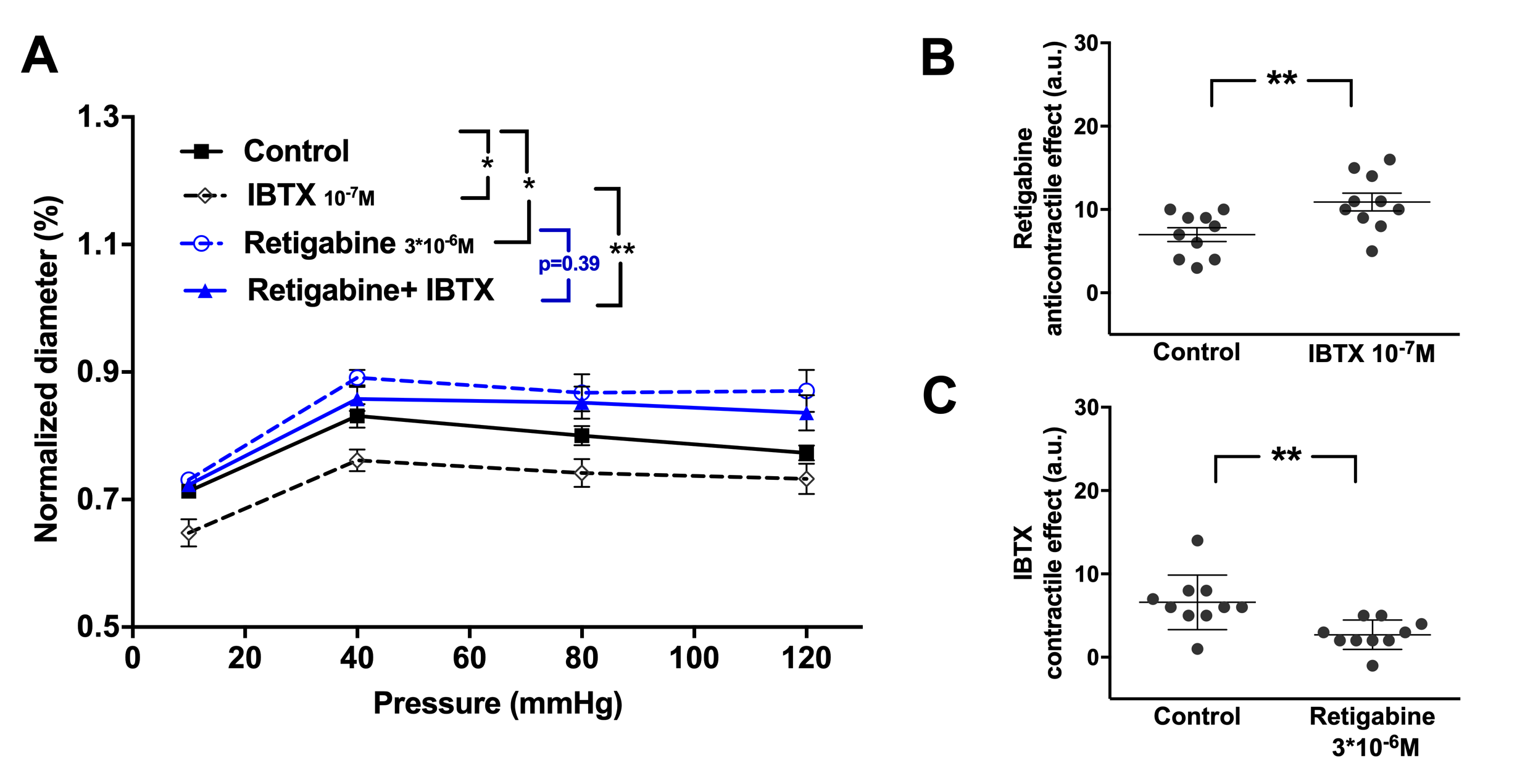

Supplement: Supplementary Figure 6 — Effect of retigabine and IBTX on the myogenic response of the gracilis artery. (A) Normalized diameter of gracilis arteries at different intra-luminal pressures in the absence of potassium channel active agents (Control), in the presence of IBTX (IBTX 10–7M), in the presence of retigabine (Retigabine 3∗10–6M) and in the combined presence of retigabine and IBTX (Retigabine and IBTX). (B) Retigabine anti-contractile effect in the absence (Control) and presence of IBTX (IBTX 10–7M). (C) IBTX contractile effect in the absence (Control) and presence of retigabine (Retigabine 3∗10–6M). n = 10; ∗p < 0.05, ∗∗p < 0.01. [file Image_6.TIFF]

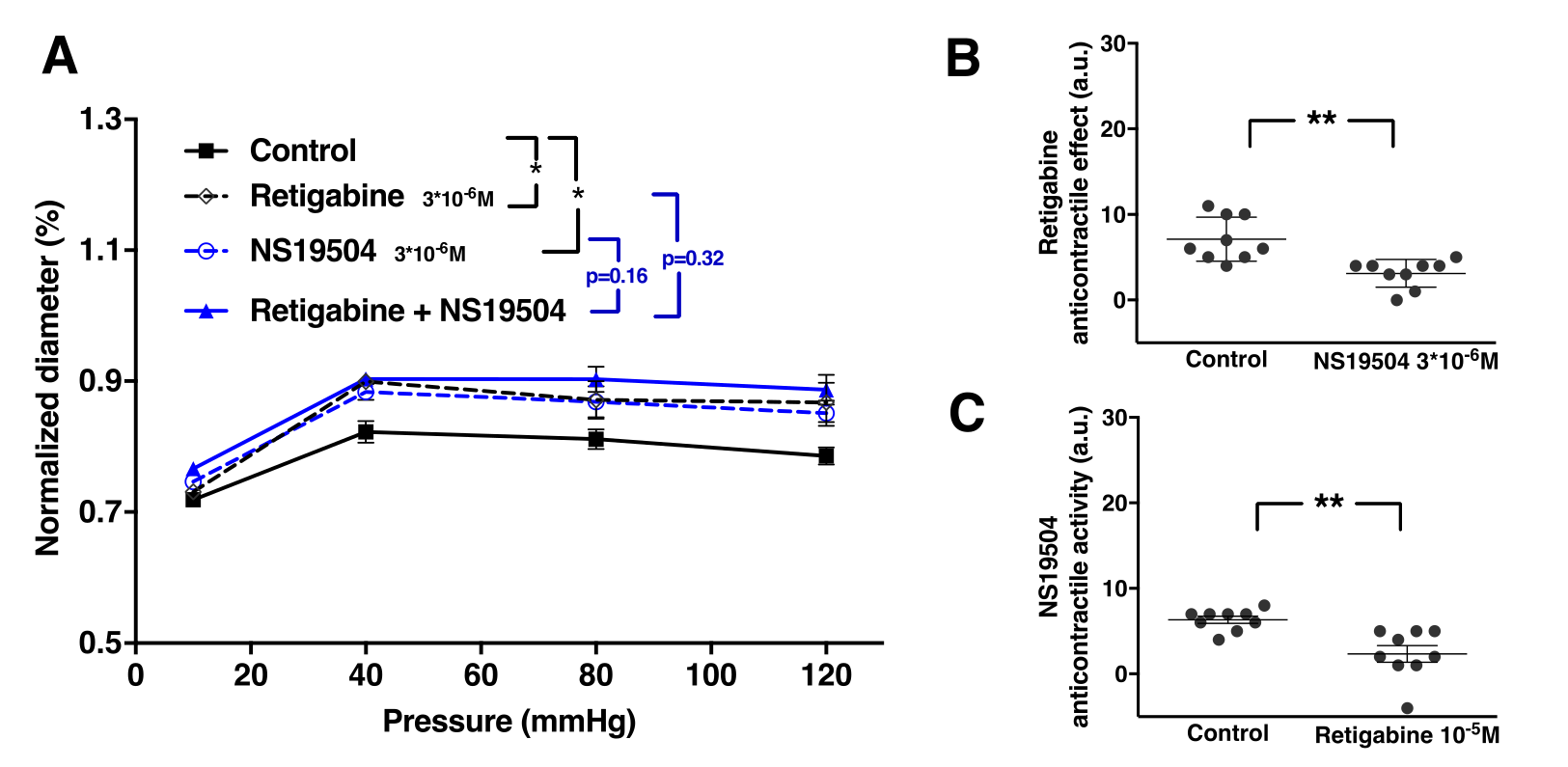

Supplement: Supplementary Figure 7 — Effect of retigabine and NS19504 on the myogenic response of the gracilis artery. (A) Normalized diameter of gracilis arteries at different intra-luminal pressures in the absence of potassium channel active agents (Control), in the presence of retigabine (Retigabine 3∗10–6M), in the presence of NS19504 (NS19504 3∗10–6M) and in the combined presence of retigabine and NS19504 (Retigabine + NS19504). (B) Retigabine anti-contractile effect in the absence of (Control) and presence of NS19504 (NS19504 3∗10–6M). (C) NS19504 anti-contractile effect in the absence of (Control) and presence of retigabine (Retigabine 3∗10–6M). n = 9; ∗p < 0.05, ∗∗p < 0.01. [file Image_7.TIFF]

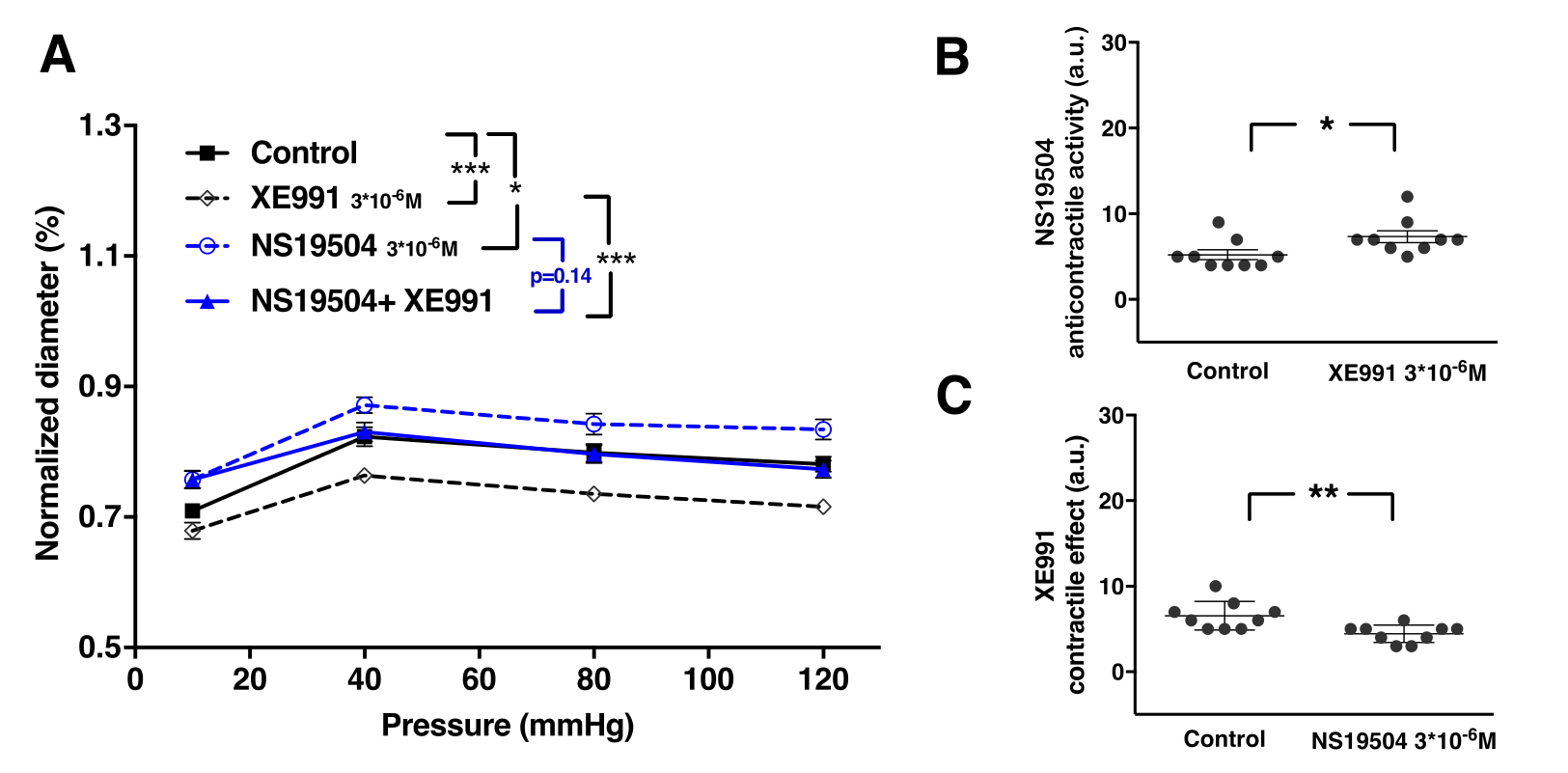

Supplement: Supplementary Figure 8 — Effect of NS19504 and XE991 on the myogenic response of the gracilis artery. (A) Normalized diameter of gracilis arteries at different intra-luminal pressures in the absence of potassium channel active agents (Control), in the presence of XE991 (XE991 3∗10–6M), in the presence of NS19504 (NS19504 3∗10–6M) and in the combined presence of NS19504 and XE991 (NS19504 + XE991). (B) NS19504 anti-contractile effect in the absence (Control) and presence of XE991 (XE991 3∗10–6M). (C) XE991 contractile effect in the absence (Control) and presence of NS19504 (NS19504 3∗10–6M). n = 9; ∗p < 0.05, ∗∗p < 0.01; ∗∗∗p < 0.001. [file Image_8.TIFF]

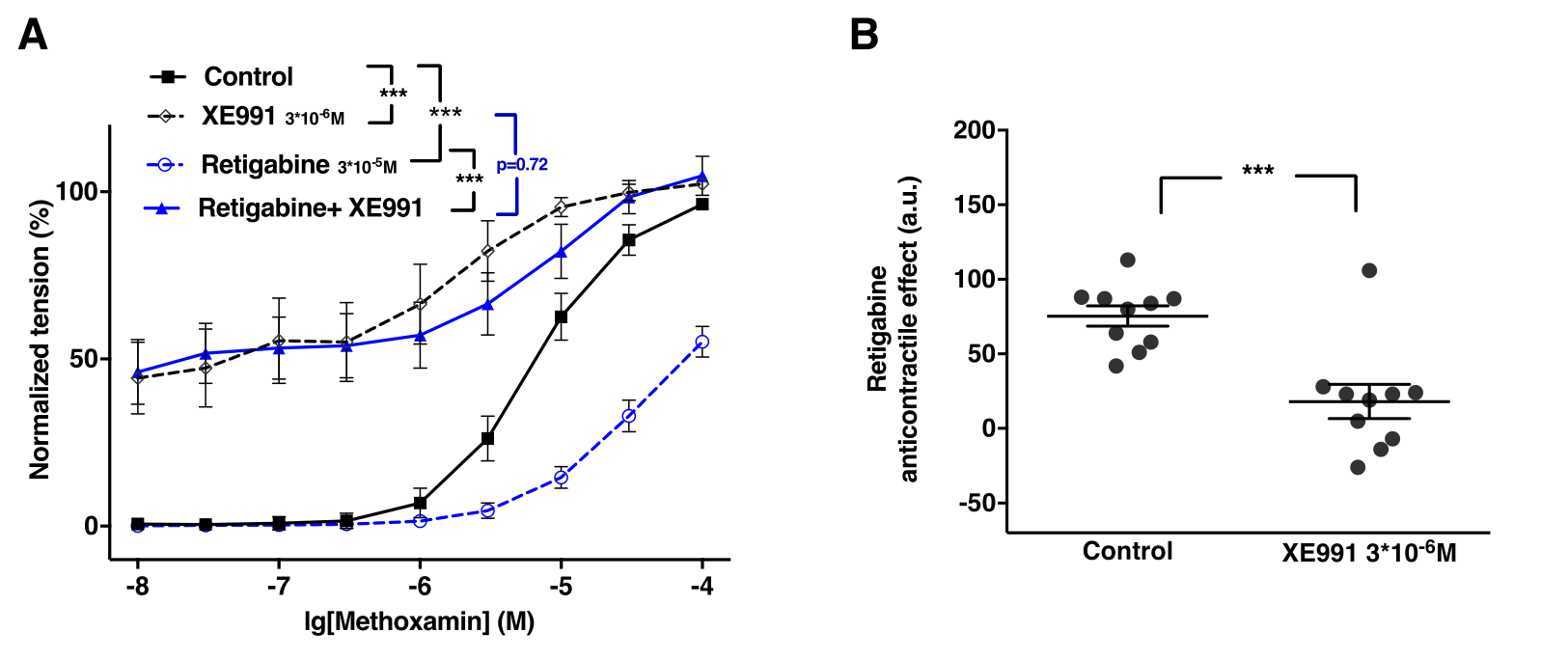

Supplement: Supplementary Figure 9 — Effect of retigabine and XE991 on methoxamine-induced contractions of the saphenous artery of young rats. (A) Normalized tension of saphenous arteries at different methoxamine concentrations in the absence of Kv7 channel active agents (Control), in the presence of XE991 (XE991 3∗10–6M), in the presence of retigabine (Retigabine 3∗10–5M) and in the combined presence of retigabine and XE991 (Retigabine + XE991). (B) Anti-contractile effect of retigabine in the absence (Control) and presence of XE991 (XE991 3∗10–6M). n = 10; ∗∗∗p < 0.001. [file Image_9.TIFF]

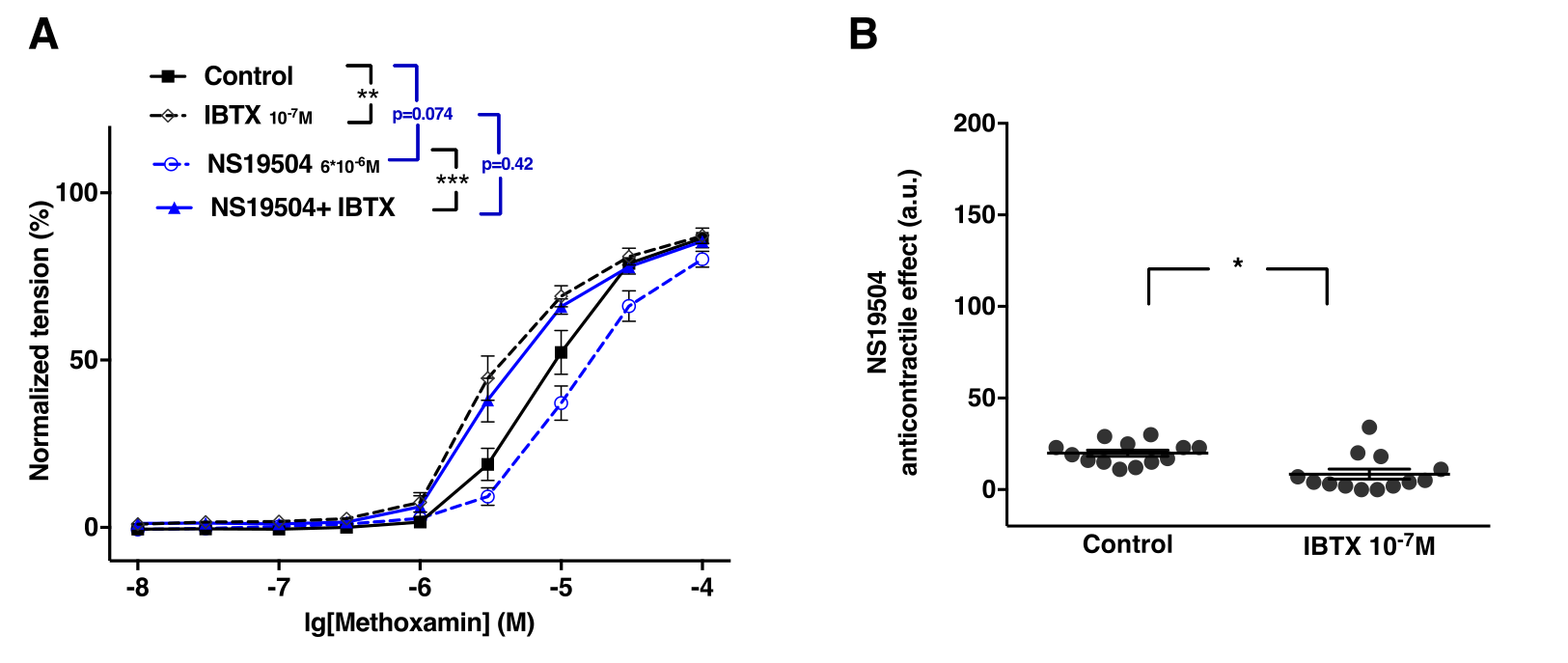

Supplement: Supplementary Figure 10 — Effect of NS19504 and IBTX on methoxamine-induced contractions of the saphenous artery of young rats. (A) Normalized tension of saphenous arteries with different methoxamine concentrations in the absence of BK channel active agents (Control), in the presence of IBTX (IBTX 10–7M), in the presence of NS19504 (NS19504 6∗10–6M) and in the combined presence of NS19504 and IBTX (NS19504 and IBTX). (B) NS19504 anti-contractile effect in the absence (Control) and presence of IBTX (IBTX 10–7M). n = 13; ∗p < 0.05, ∗∗p < 0.01; ∗∗∗p < 0.001. [file Image_10.TIFF]
